# Mechanochemical Preparation of Dipyridyl-Naphthalenediimide Cocrystals: Relative Role of Halogen-Bond and π–π Interactions

**DOI:** 10.1021/acs.cgd.1c00531

**Published:** 2021-09-01

**Authors:** Paolo P. Mazzeo, Marianna Pioli, Fabio Montisci, Alessia Bacchi, Paolo Pelagatti

**Affiliations:** † Dipartimento di Scienze Chimiche, della Vita e della Sostenibilità Ambientale, Università di Parma, Parco Area delle Scienze 17/A, 43124 Parma, Italy; ‡ Biopharmanet-TEC, Università di Parma, Parco Area delle Scienze 27/A, 43124 Parma, Italy; § Centro Interuniversitario di Reattività Chimica e Catalisi (CIRCC), Via Celso Ulpiani 27, 70126 Bari, Italy

## Abstract

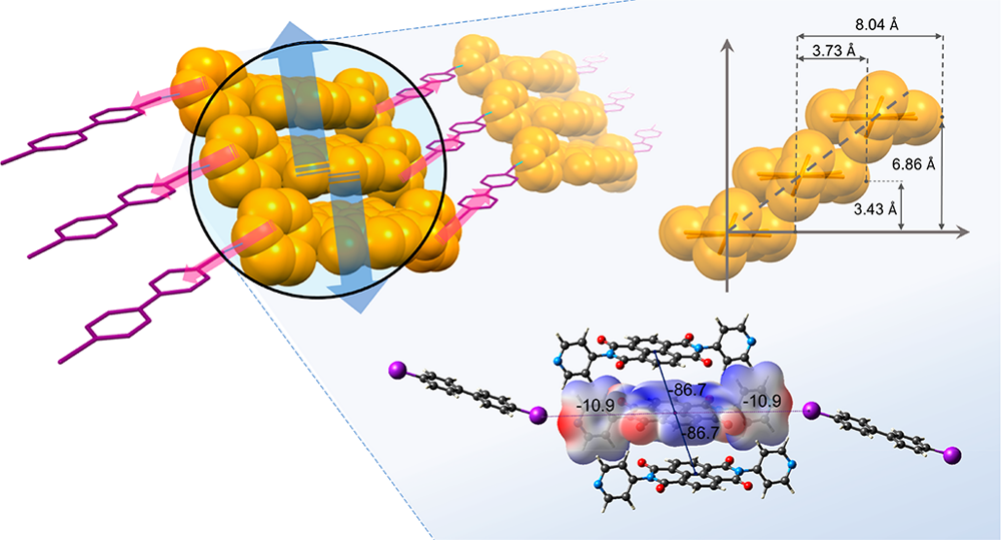

Naphthalenediimide derivates are a class of π-conjugated molecules largely investigated in the literature and used as building blocks for metal–organic frameworks or coformers for hydrogen-bond-based cocrystals. However, their tendency to establish halogen-bond interactions remains unexplored. By using a crystalline engineering approach, we report here four new cocrystals with *N*,*N*′-di(4-pyrydyl)-naphthalene-1,4,5,8-tetracarboxidiimide and diiodo-substituted coformers, easily obtained via a mechanochemical protocol. Cocrystals were characterized via NMR, electron ionization mass spectrometry, thermogravimetric analysis, powder X-ray diffraction, and single-crystal X-ray diffraction. Crystallographic structures were then finely examined and correlated with energy framework calculations to understand the relative contribution of halogen-bond and π–π interactions toward framework stabilization.

## Abstract 2

Mechanochemical synthesis allows the synthetic problems deriving from low solubility of a py-functionalized naphthalenediimide in the synthesis of halogen-bond cocrystals with iodine-containing coformers to be overcome.

## Introduction

Cocrystals are multicomponent compounds made of different chemical entities stoichiometrically interacting within the crystal lattice.^1−4^ Cocrystallization alters the physical–chemical properties of the individual molecular components; designing a cocrystal requires a thorough knowledge of the possible intermolecular affinity between the molecular partners, providing a robust intermolecular network.^4^ In fact, even though cocrystals have been extensively studied in the framework of crystal engineering,^5,6^ their use is still mainly centered within the pharmaceutical arena,^7^ although some interesting environmental-related studies have recently appeared in the literature.^3,8−10^

**Scheme 1:**
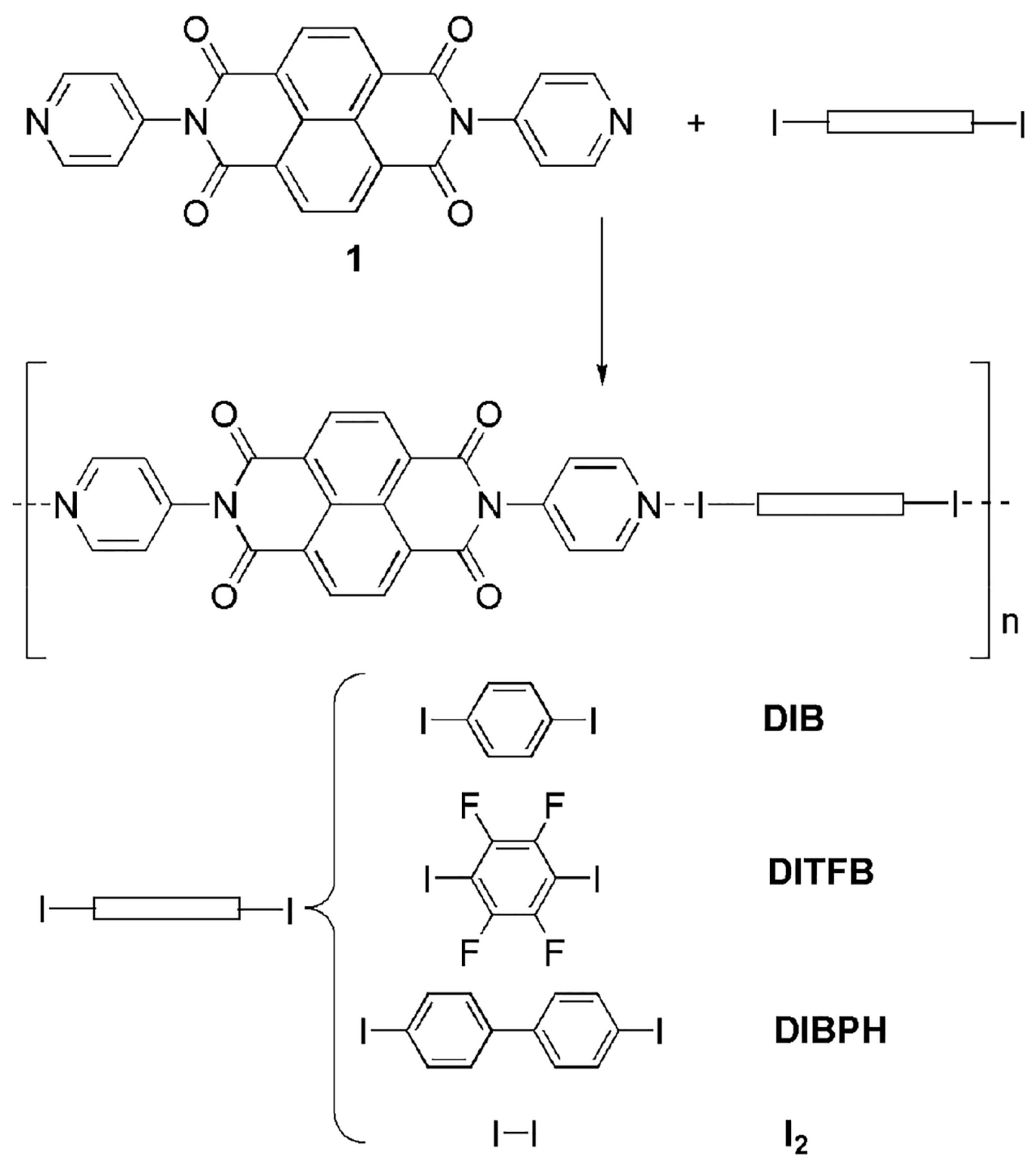
General Scheme of the Cocrystals Investigated

*N*,*N*′-di(4-pyridyl)-naphthalene-1,4,5,8-tetracarboxydiimide (**1**) belongs to the class of naphthalenediimides (NDI), rigid π-conjugated molecules characterized by an electron-poor naphthalene core (Scheme 1) largely investigated in the past decade. Their electron affinity, ability to behave as charge carriers, and excellent thermal and oxidative stability make them promising candidates for organic electronic applications, photovoltaic devices, and flexible displays.^11−15^ The robustness of the aromatic core has pushed forward the use of NDIs as rigid linkers for chemoresponsive luminescent metal–organic frameworks (MOFs),^16−22^ metallacycles,^23−26^ or supramolecular assemblies.^27^ Exploiting their affinity with aromatic guest molecules that ultimately influence their emission profile, it is possible to reveal the guest uptake even at very low concentrations.^19^ NDIs have also been largely investigated as coformers for hydrogen-bond (HB)-based cocrystals.^28^ Although pyridine-based systems have been extensively used for halogen-bond (XB)-based cocrystals,^29^ to the best of our knowledge **1** has never been embedded in a cocrystal matrix through a halogen bond connecting the pyridine moieties with XB donors. To explore the ability of **1** as an XB acceptor in cocrystals, we performed a series of cocrystallization experiments between **1** and several diiodo-substituted organic molecules, such as 1,4-diiodobenzene (**DIB**), 1,4-diiodotetrafluorobenzene (**DITFB**), 4,4′-diiodobiphenylene (**DIBPH**), and molecular iodine (**I**_**2**_), as depicted in Scheme 1. The halogen-bond interaction should lead to the formation of 1D chains supported by halogen-bond intermolecular interactions, where the pyridine nitrogen atoms act as halogen-bond acceptors, while the iodine atoms play the role of the halogen-bond donors. Owing to the low solubility of **1**, cocrystallization reactions conducted in solution led to the isolation of X-ray quality single crystals of the target compounds in poor yields. To overcome this problem, the synthesis of the target cocrystals was attempted by manual grinding of **1** with the corresponding halogenated coformers, in the presence of substoichiometric amounts of DMF (LAG = liquid-assisted grinding). It is well-known that mechanochemical synthesis is often able to overcome the problems derived from the use of insoluble reagents, and its successful application in the preparation of cocrystals includes a large number of literature reported examples.^30,31^ Here we then demonstrate that halogen-bond cocrystals derived from the combination of the poorly soluble π-donor **1** with four different iodine-containing coformers can be synthesized in high purity through a simple mechanochemical approach. The new crystalline compounds were characterized by analytical, thermal, and spectroscopic techniques, and their solid-state structures were solved by single-crystal X-ray diffraction correlated with energy framework calculations to highlight the nature of the stabilizing intermolecular interactions.

## Experimental Section

### Materials

1,4,5,8-Naphthalenetetracarboxylic dianhydride, 4-aminopyridine, as well as all the diiodo-substituted organic coformers were purchased from Sigma Aldrich and used as such without any further purification. DMF was stored over activated 5 Å molecular sieves under a nitrogen atmosphere.

### Instruments

#### Melting Point

Melting points of the four cocrystals were determined by means of a Gallenkamp melting point apparatus equipped with a digital thermometer. Crystalline samples were gently ground, and a few milligrams was packed inside an open borosilicate glass capillary which was inserted in the heating chamber. Heating was conducted with a temperature ramp of 2 °C/min (±0.1 °C resolution), in the temperature range 25–330 °C. Check of the physical changes undergone by the sample was done through a viewing hole mounting a magnifying length.

#### Fourier Transform Infrared-Attenuated Total Reflectance

FTIR-ATR spectra were acquired by means of a Nicolet-Nexus ThermoFisher spectrophotometer equipped with a diamond ATR crystal, in the frequency region 4000–400 cm^–1^.

#### Nuclear Magnetic Resonance

^1^HNMR spectra were acquired by means of a Bruker AV300 or AV400 spectrophotometer, using a mixture of DMSO-*d*_6_ and CF_3_COOD (one drop) to facilitate the complete solubilization of the solids. The ^19^F{^1^H}-NMR spectrum of **1** was instead recorded in DMSO-*d*_6_ after prolonged sonication. The ^1^H chemical shift values are referenced to TMS, while the ^19^F{^1^H} chemical shift is referenced to CFCl_3_. Elemental analyses were performed on a FlashEA 1112 Series CHNS-O analyzer (ThermoFisher) with gas-chromatographic separation.

#### Mass Spectrometry

MS-EI(+) analyses were performed on a ThermoScientific DSQII with a direct insertion probe (DIP) for direct loading of solid samples. The crystalline sample (less than 1 mg) of the product was placed in a microvial that was inserted into the tip of the DIP probe. After introduction of the probe into the spectrometer, the temperature of the probe was raised with a temperature ramp of 10 °C/min, until a clear spectrum of the organic iodide was obtained. The very low vapor tension of NDI prevents contamination of the spectrum with its own signals leading, in the case of cocrystals, to clear spectra of the corresponding organic iodides.

#### Thermal Analyses

Thermogravimetric analysis (TGA) was performed on a PerkinElmer TGA7 apparatus (Pt-crucible) typically in the range 30–400 °C at the heating rate of 10 °C/min with a constant purge of dry nitrogen (see Supporting Information).

#### Powder X-ray Diffraction (PXRD)

Data collection were performed with a Thermo ARL X’TRA powder diffractometer equipped with a Thermo Electron solid-state detector with CuKα radiation in Bragg–Brentano geometry.

#### Single-Crystal X-ray Diffraction (SCXRD)

Data collection was performed on a Bruker Smart diffractometer equipped with an Apex II CMOS detector and a sealed tube Mo source (λ = 0.71073 Å). The collected intensities were corrected for Lorentz and polarization factors and empirically for absorption by using the SADABS program.^32^ Structures were solved using SHELXT^33^ and refined by full-matrix least-squares on all F^2^ using SHELXL^34^ implemented in the Olex2 package.^35^ Hydrogen atoms were added in calculated positions. Anisotropic displacement parameters were refined for all non-hydrogen atoms. Table 2 summarizes crystal data and structure determination results. Crystallographic data have been deposited with the Cambridge Crystallographic Data Centre (CCDC deposition numbers: 2079372–2079375). Copies of the data can be obtained free of charge on application to CCDC, 12 Union Road, Cambridge CB2 1EZ, UK (fax: (+44) 1223-336-033; e-mail: deposit@ccdc.cam.ac.uk).

### Synthesis of **1**

*N*,*N*′-di(4-pyridyl)-naphthalene-1,4,5,8-tetracarboxydiimide (**1**) was synthesized following a slightly modified procedure with respect to the method reported in the literature:^23^ 1,4,5,8-naphthalene-tetracarboxydianhydride (500 mg, 1.886 mmol) and 4-aminopyridine (350 mg, 3.752 mmol) were placed in a 100 mL two-necked round-bottom flask with 15 mL of anhydrous DMF. The sample was stirred and purged with nitrogen gas. The mixture was then refluxed under a nitrogen atmosphere at 130 °C for 18 h. The homogeneous brown solution obtained was cooled down at room temperature. The resulting whitish powder precipitate was filtered on a Büchner funnel, washed with fresh DMF and diethyl ether, and then oven-dried at 110 °C for 60 min. Yield: 64% (502 mg); mp > 300 °C. IR-ATR: n (cm^–1^) 3034, 1717, 1667, 1576, 1350. ^1^H NMR (DMSO-*d*_6_): d 8.78 (d, 4H, py), 8.71 (s, 4H, napht.), 7.55 (d, 4H, py).

### General Procedures for the Synthesis of the Cocrystals

#### Solution Syntheses

Reaction between **1** with the XB donor (XB: halogen bond) in hot DMF led to the isolation of nicely faceted crystals corresponding to the target cocrystal products. The reactions were carried out at 130 °C, thus having **1** completely dissolved in DMF. In all cases, the occurrence of the reactions was indicated by a color change of the solutions. The uncolored starting DMF solution of free **1** became orange after the addition of **DIB** or **DIBPH** and purple after the addition of **DITFB** or **I**_**2**_. The reactant solutions were kept under stirring and reflux for 1 h and then slowly cooled to room temperature. In all cases, during the cooling process, part of **1** precipitated as a beige solid (amounts not quantified), as confirmed by FTIR analysis (see Supporting Information). The remaining clear solutions were left to slowly evaporate to room temperature. Needle-like crystals suitable for SCXRD analysis were then collected. The yield of cocrystallization was then referred to the amount of X-ray quality single crystals collected from clear solutions. The characterization data regarding elemental analysis, ^1^H NMR, EI-MS, and TGA referred to the crystalline materials.

##### **1-DIB**

**1** (100 mg, 0.238 mmol) and **DIB** (78 mg, 0.238 mmol) were placed in 40 mL of DMF at 130 °C under stirring. Orange crystals of the titled compound were filtered by the orange solution and analyzed by SCXRD. Yield: 20% (36 mg); mp > 330 °C (crystals degradation at about 200 °C); elemental analysis for C_30_H_16_I_2_N_4_O_4_ (found): C, 48.02 (48.00); H, 2.15 (2.05); N, 7.47 (7.48); ^1^H NMR (DMSO-*d*_6_/CF_3_COOD): d 9.19 (d_br_, 4H, py), 8.79 (s, 4H, napht.), 8.31 (d, 4H, py), 7.44 (4H, DIB); MS-EI(+) DIP (probe temperature: 40 °C): *m*/*z* = 329.8 [C_6_H_4_I_2_]^+^; TGA (temperature range: 30–400 °C, 10 °C/min): observed loss (expected): 43.6% (44%); T interval: 90–220 °C.

##### **1-DIBPH**

**1** (44.27 mg, 0.238 mmol) and **DIBPH** (483 mg, 1.190 mmol) were placed in 40 mL of DMF at 130 °C under stirring. Orange crystals of the titled compound were filtered by the orange solution and analyzed by SCXRD. Yield: 21% (41.3 mg); mp >330 °C (crystals degradation at 278–279 °C); elemental analysis for C_36_H_20_I_2_N_4_O_4_ (found): C, 52.32 (51.98); H, 2.44 (2.40); N, 6.78 (6.99); ^1^H NMR (CDCl_3_/CF_3_COOD): d 9.23 (d, 4H, py), 8.81 (s, 4H, napht.), 8.32 (d, 4H, py), 7.80 (d, 4H, DIBPH), 7.44 (d, 4H, DIBPH); MS-EI(+) DIP (probe temperature: 50 °C): *m*/*z* = 405 [C_12_H_8_I_2_]^+^; TGA (temperature range 25–400 °C, 5 °C/min): observed loss (expected) 46.4% (49.1%).

##### **1-DITFB**

**1** (100 mg, 0.238 mmol) and **DITFB** (101.9 mg, 0.476 mmol) were placed in 40 mL of DMF at 130 °C under stirring. Orange crystals of the titled compound were filtered by the purple solution and analyzed by SCXRD. Yield: 6% (11.7 mg): mp >330 °C (crystal degradation at about 230 °C); elemental analysis for C_30_H_12_F_4_I_2_N_4_O_4_ (found): C, 43.82 (43.68); H, 1.47 (1.55); N, 6.81 (6.68); ^1^H NMR (DMSO-*d*_6_/CF_3_COOD): equivalent to that of **1**. ^19^F{^1^H} NMR: d 76.49 (s); MS-EI(+) DIP (probe temperature: 150 °C): *m*/*z* = 401.7 [C_6_F_4_I_2_]^+^; TGA (temperature range: 25–400 °C, 10 °C/min): observed loss (expected): 45.3% (48.9%).

##### **1-I**_**2**_

**1** (100 mg, 0.238 mmol) and I_2_ (121 mg, 0.476 mmol) were placed in 40 mL of DMF at 130 °C under stirring. Orange crystals of the title compound were filtered by the purple solution and analyzed by SCXRD. Yield: 34% (54.6 mg); mp >330 °C (crystal opacification at 220 °C); elemental analysis for C_24_H_12_I_2_N_4_O_4_ (found): C, 42.76 (42.81); H, 1.79 (1.98); N, 8.31 (8.28); ^1^H NMR (DMSO-*d*_6_/CF_3_COOD): equivalent to that of **1**. MS-EI(+) DIP (probe temperature: 150 °C): *m*/*z* = 253.6 [I_2_]^+^. TGA (temperature range: 25–400 °C, 10 °C/min): observed loss (expected): 34% (37.6%).

#### Mechanochemical Syntheses

Bulk powders were prepared by liquid-assisted grinding (LAG): equimolar amounts of **1** (50 mg, 0.119 mmol) and XB donor were manually ground in an agate mortar together with 50 μL of DMF. The grinding was maintained for about 60 min, with regular interruptions to recollect the solids from the mortar walls and pestle. Already at the initial stages of the grinding, the solid mixtures assumed different colors according to the XB donor, which became more and more intense as a function of time. In all cases, PXRD analyses performed on the final samples were in agreement with those calculated from SCXRD structures.

**1-DIB**: DIB, 39 mg; the product appears as a microcrystalline yellow powder.

**1-DIBPH**: DIBPH, 30.7 mg; the product appears as a microcrystalline crimson powder.

**1-DITFB**: DITFB, 21.7 mg; the product appears as a microcrystalline ochre powder.

**1-I**_**2**_: I_2_, 12.6 mg; the product appears as a microcrystalline yellow powder.

### Computational Methods

Estimation of the intermolecular interaction energies and energy frameworks was performed with CrystalExplorer17^36,37^ at the HF/3-21G level of theory (for a cluster of 5 Å around each molecule in the asymmetric unit).^37^ Molecular electrostatic potential (MEP) was calculated with Tonto^38^ using density functional theory at the B3LYP/6-311G(d,p) level and displayed using CrystalExplorer17; MEP was mapped on the electron density surface cut at the 0.002 au level. Dispersive stabilization in all cocrystals structures was also ranked according to the aromatic analyzer tool recently implemented in the CCDC CSD-Material Suite.^39^

## Results and Discussion

### Synthesis

The first synthetic attempts were based on a wet procedure. **1** is poorly soluble in many organic solvents, while it dissolves in hot DMF. The syntheses were then initially conducted in such a solvent at 130 °C using an excess of the XB donor. The clear solutions were then slowly cooled to room temperature to allow crystallization. Although the thermal reaction led to the target compounds as X-ray quality single crystals, the final yields were highly unsatisfactory (not higher than 34%) owing to the precipitation of part of **1** during the initial stages of cooling. To overcome this drawback, we decided to investigate the possibility of isolating the target compounds by mechanochemistry, a technique that often allows circumventing the problems derived from the insolubility of the reactants.^40^ Manual grinding is a very simple approach which allows the use of a mortar and a pestle by which the coformers are manually ground. Neat grinding and liquid-assisted grinding (LAG) have extensively been used for the preparation of a large number of pyridine-containing cocrystals.^41^ Neat grinding is conducted in the absence of liquid, while LAG considers the use of substoichiometric amounts of a liquid. LAG is often described to speed up cocrystal formation. On the basis of these considerations, we approached the synthesis of the four target cocrystals by the LAG procedure. All the reactions were conducted starting from 50 mg of **1**, the required amount of XB donor to satisfy a 1:1 molar ratio and 50 μL of DMF. An agate mortar and pestle were used, and the progress of the reaction was monitored by PXRD analysis. The nearly complete conversion of the reagents into the target cocrystals was achieved within 60 min of grinding, based on the comparison of the experimental and calculated PXRD traces (see Supporting Information). This confirmed the effectiveness of the simple mechanochemical approach adopted for the synthesis of the four new cocrystal compounds.

### Chemical Characterization

Before being subjected to structural analysis, the crystals collected from DMF were investigated by elemental analyses, NMR spectroscopy, and TGA analysis to ensure the presence of the iodine-containing coformer. The elemental analyses confirmed the expected stoichiometries, indicating a 1:1 ratio between **1** and the corresponding coformers. To ensure a complete crystal solubilization, the NMR spectra were collected in DMSO-*d*_6_ with one drop added of CF_3_COOD. As expected, the spectra of **1-DIB** and **1-DIBPH** corresponded to the sum of the spectra of **1** and the corresponding coformer (see Supporting Information). However, the ratio between the integration areas of the aromatic protons of **1** and those of the coformer was indicative of a 1:1 ratio between the two components. The ^1^H NMR spectra **1-DITFB** and **1-I**_**2**_ were not recorded because of the absence of protons in the halogenated coformers. However, the presence of 1,4-diiodo-tetrafluorobenzene was confirmed by ^19^F{^1^H}NMR spectroscopy, with a singlet at −120.25 ppm (see Supporting Information). The presence in the crystals of the halogenated coformer was further confirmed by DIP-EI(+) MS analysis, which allows the MS analysis of the analytes thermally extruded from the crystals (see Experimental Section for details). Since the volatility of **1** is much lower than that of the four coformers, clear mass spectra of DIB, DIBPH, DITFB, and I_2_ were collected. In all spectra, the most intense peak was that of the ionized halogenated coformers, with signals at *m*/*z* values of 329.8, 405.8, 401.7, and 253.6 for **1-DIB**, **1-DIBPH**, **1-DITFB**, and **1-I**_**2**_, respectively. The other signals were in agreement with the expected fragmentation patterns (see Supporting Information). TGA analyses showed mass weight losses compatible with the thermally induced departure of the halogenated coformer (see Supporting Information). In the case of **1-DIB**, the main weight loss corresponding to 43.6% was observed in the interval 90–220 °C (expected value: 44%), although the loss extends up to about 280 °C. In the case of **1-DIBPH**, the coformer extrusion occurred at a higher temperature, in accordance with the higher melting point of DIBPH (201–204 °C) with respect to DIB (131–133 °C). The weight loss corresponding to 46.4% was in fact observed in the interval 150–290 °C (expected value 49.1%), although the loss extended up to 400 °C. In the case of **1-DITFB**, although the perhalogenated coformer has a lower melting point with respect to DIB, the main weight loss corresponding to 45.3% (expected value 48.9%) started at 150 °C and was completed at 290 °C, where a second weight loss corresponding to 4.3% started and was completed within 400 °C. A total weight loss of 48.3% was then recorded, in perfect agreement with the expected value. Finally, in the case of **1-I**_**2**_, the high volatility of iodine leads to a detectable weight loss already in the initial stages of the analysis, although the main loss corresponding to 34% (expected value 37.6%) was observed in the interval 200–290 °C. Visual inspection during the recording of the melting points evidenced crystal deterioration at temperatures close to those corresponding to the main weight loss percentages found in the TGA traces, likely due to the thermally induced XB donor departure. Prolonging the heating up to 330 °C left a solid residue in the capillary, that we assume to correspond to **1**, whose melting point is higher than 400 °C.^11^

### Structural Analysis

**Figure 1:**
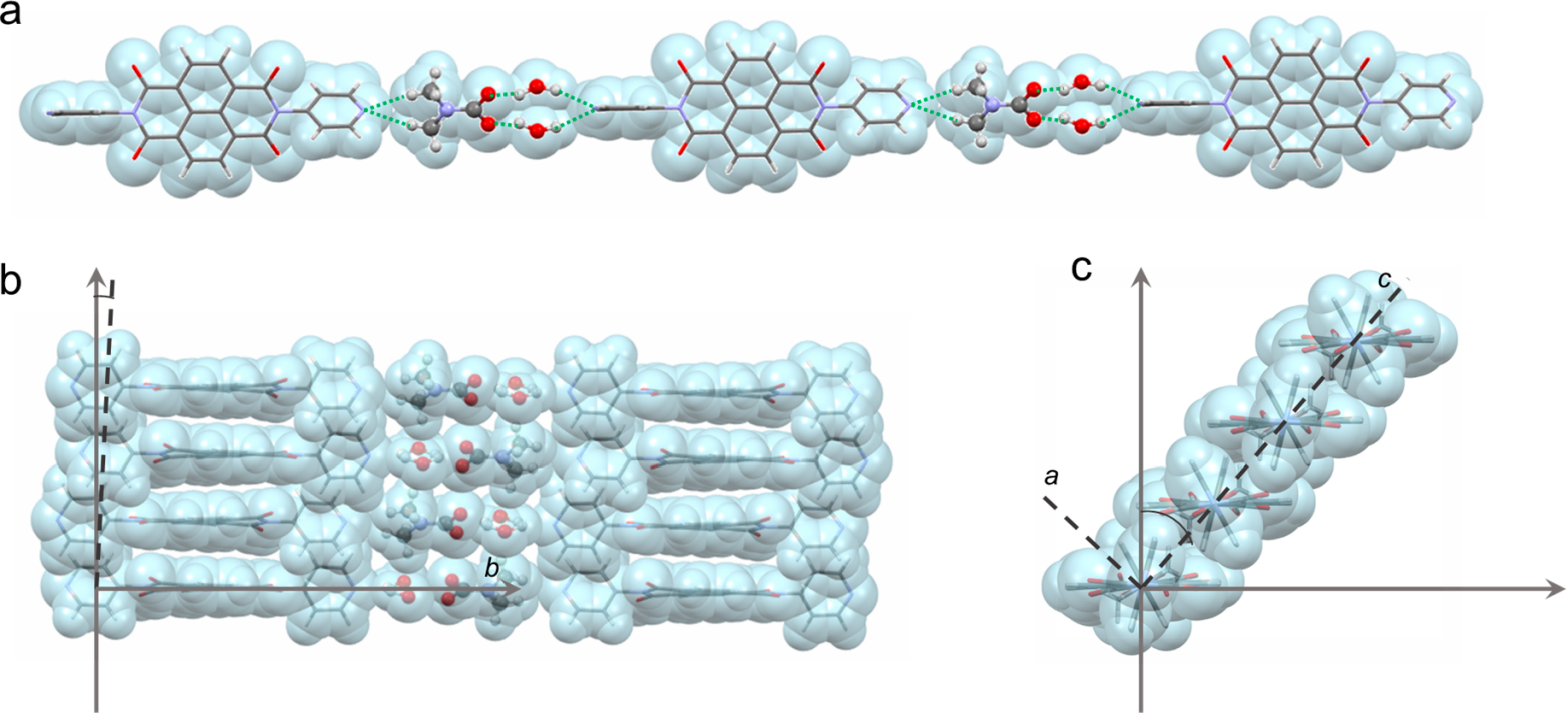
(a) Representation of the hydrogen-bond 1D chain in **1-DMF-2H**_**2**_**O** along the *b*-axis. Solvent molecules are reported in ball and stick style. DMF molecules are disordered into two mutually exclusive orientations. Hydrogen bonds are highlighted by green dashed lines. Pitch angle (b) and roll angle (c) observed along the *b*-axis.

In the recent literature, **1** has never been reported as an anhydrous/unsolvate form, while different solvate structures of **1** are, instead, described.^21,42−44^ In fact, we observed the solvate hydrate form **1-DMF-2H**_**2**_**O**, already reported by Lin et al.,^44^ as a concomitant product from the cocrystallization experiment. For the reader’s convenience, a brief structural description of **1-DMF-2H**_**2**_**O** is reported here. The DMF molecules are disordered into two mutually exclusive positions in which the oxygen atom of the carbonyl group is differently oriented. Two water molecules bridge the DMF to **1** forming a *D*_3_^2^ (6) hydrogen-bond graph set (Figure 1).

**Figure 2:**
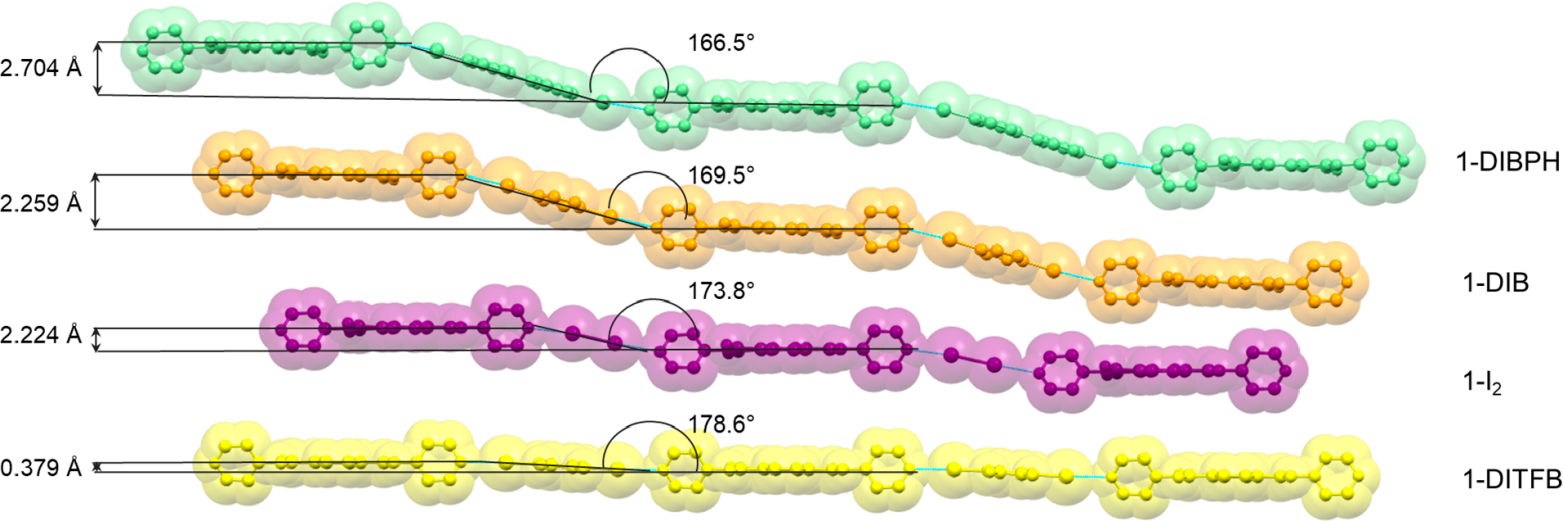
Structure overlay of **1-DIBPH** (green), **1-DIB** (orange), **1-I**_**2**_ (purple), **1-DITFB** (yellow), along the naphthalene aromatic moieties. Interplanar distances between two consecutive NDIs belonging to the same chain are reported as well. Angles between mean planes describing the coformer molecular cores are also reported.

The observed chain motif for the solvate form can be contrasted with the cocrystal structures reported in our work, which show a recurrent XB network with a motif, as reported in Figure 2. We evidence two main structural factors describing this recurrent motif: the halogen-bond angles are a function of the XB donor used, and the distance between NDI molecules within the same polymeric chain increases with an increase of the XB donor molecular length and with a decrease of the XB angle.

These trends have been investigated by structural analysis and energy considerations. The structural motif of XB-based cocrystals strongly depends on the halogen-bond distances and angles, as reported by Brammer et al.^45^ A statistical analysis performed on the CSD database returned a correlation trend of the distribution of I···N(py) distances in crystal structures containing molecular iodine or iodo-substituted organic molecules as a function of the halogen-bond angle as shown in Figure S11 and reported in Table 1. In the case of linear orientation, the average halogen-bond distance is 2.816(3) Å, which increases significantly at 3.79(2) Å in the case of bent orientation.

**Table 1:** Halogen-Bond (XB) Distances and Angles Observed in **1-I**_**2**_, **1-DIB**, **1-DITFB**, and **1-DIBPH**^a^

	**1-I**_**2**_	**1-DIB**	**1-DITFB**	**1-DIBPH**
XB distance (Å)	2.86^b^	3.07	2.83^b^	3.09
XB angle (deg)	176^b^	175.5	178^b^	174
pitch angle_1,2_ (deg)	1.98	0.00	1.47	0.00
pitch angle_1,3_ (deg)	2.36	0.74	0.85	0.90
roll angle_1,2_ (deg)	39.90	47.43	43.87	46.21
roll angle_1,3_ (deg)	40.92	49.53	46.91	48.36
*d*_1,2_ (Å)	3.36	3.43	3.33	3.48
*d*_1,3_ (Å)	6.86	6.86	6.62	6.96
*d*_P1,2_ (Å)	0.12	0.00	0.09	0.00
*d*_P1,3_ (Å)	0.28	0.09	0.10	0.11
*d*_R1,2_ (Å)	2.81	3.73	3.20	3.63
*d*_R1,3_ (Å)	5.94	8.04	7.08	7.83
*D*_off1,2_ (Å)	2.82	3.73	3.21	3.63
*D*_off1,2_ (Å)	5.95	8.04	7.08	7.83

a Pitch and roll angles and calculated shift distances for the NDI π-stacking system are also reported. A schematic representation is reported in Supporting Information.

b Averaged values reported for symmetry independent molecules.

**DITFB**, as a perfluorinated molecule, is taken here as a reference to consider the ability of **1** in establishing strong halogen-bond interactions. The halogen-bond angles in **1-DITFB** are 178.0(3)° and 177.3(2) with the I···N(py) distance = 2.858(3) and 2.805(2) Å, which are respectively the widest halogen-bond angle and the shortest distance registered among the cocrystals reported here (Table 1). **1-I**_**2**_ shows a similar molecular pattern, with the XB angle of 174.0(3)° and 177.7(2)° and the XB distance of 2.826(2) and 2.887(6) Å. **1-DIB** and **1-DIBPH** show comparable halogen-bond distances (3.07(3) Å and 3.09(4) Å, respectively) and halogen-bond angles (175.5(2)° and 166.5(3)°, respectively). The energies associated with the XB interactions were estimated by CrystalExplorer17 and results are consequently quite similar (−11.1 and −10.9 kJ/mol respectively) (Tables S1–S8).

**Figure 3:**
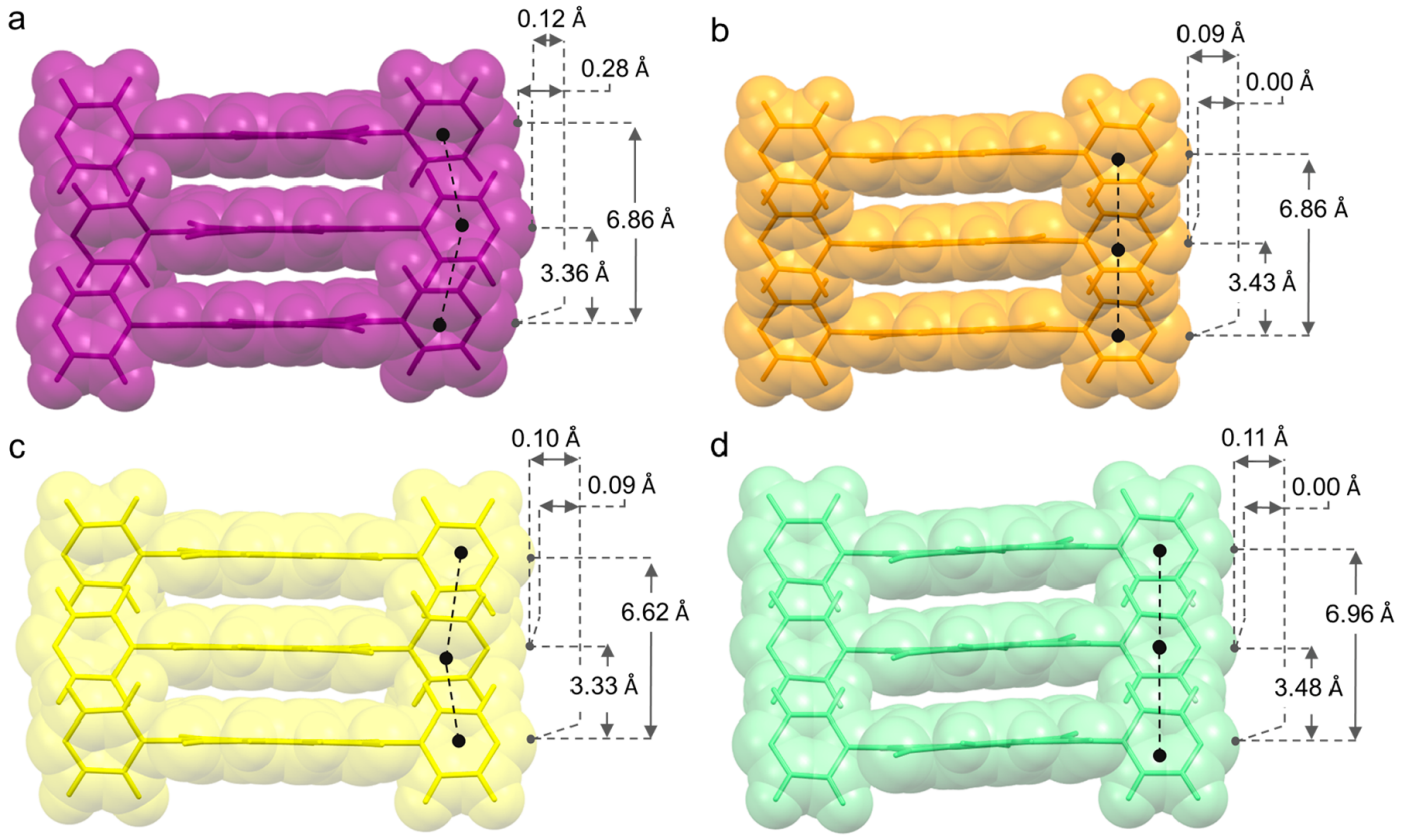
Pitch angle and pitch distance of stacked NDI molecules in **1-I**_**2**_ (a), **1-DIB** (b), **1-DITFB** (c), and **1-DIBPH** (d). Hydrogen atoms are removed for the sake of clarity.

**Figure 4:**
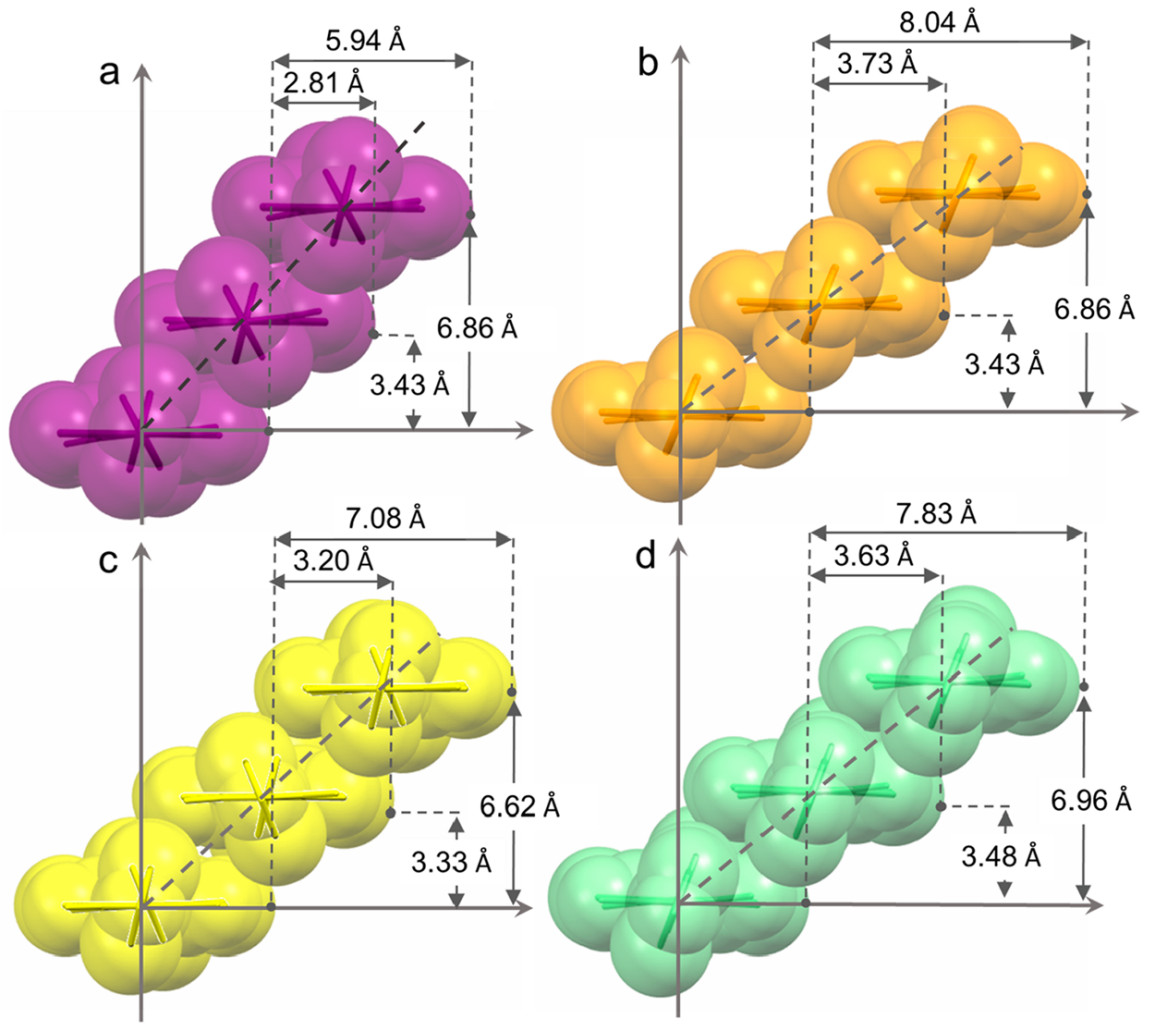
Roll angle and roll distance of stacked NDI molecules in **1-I**_**2**_ (a), **1-DIB** (b), **1-DITFB** (c), and **1-DIBPH** (d). Hydrogen atoms are removed for the sake of clarity.

Parallel to XB stabilization, we then examined the role of π–π interactions in the crystalline assembly, in order to investigate the specific role of the NDIs moiety in the energy stabilization of the cocrystals. In all cases, the naphthalene aromatic skeleton of **1** is arranged with a face-to-face motif within the crystal lattice. The appropriate way to describe the π–π interaction consists of the definition of the pitch (*P*) and roll (*R*) angles.^4,46^ The pitch distance (*d*_P_) and roll distance (*d*_R_) are consequently calculated as an orthogonal offset component respectively along the longest and shortest molecular axis. The total offset distance *D*_off_ is then calculated as follows (see also Figure S34).
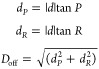
To better elucidate the piling tendency of the aromatic NDI molecules, we discriminated the pitch and roll angles and offsets between the adjacent (namely, 1 and 2) and nonadjacent (namely, 1 and 3) molecules (see Table 1, Figures 3 and 4).

Pitch distances *d*_P_ for **1-I**_**2**_ and **1-DITFB** are almost negligible being the pitch angles close to null (respectively 0.3° and 0.4°); thus, the NDI molecules are stacked with no significant translation component along the long molecular axis (Figure 3.

Roll distance, instead, largely contributes to the π-stacking motif (Table 1); thus, the NDI molecules result in being mainly offset along their shorter dimension (Figure 4.

**Figure 5:**
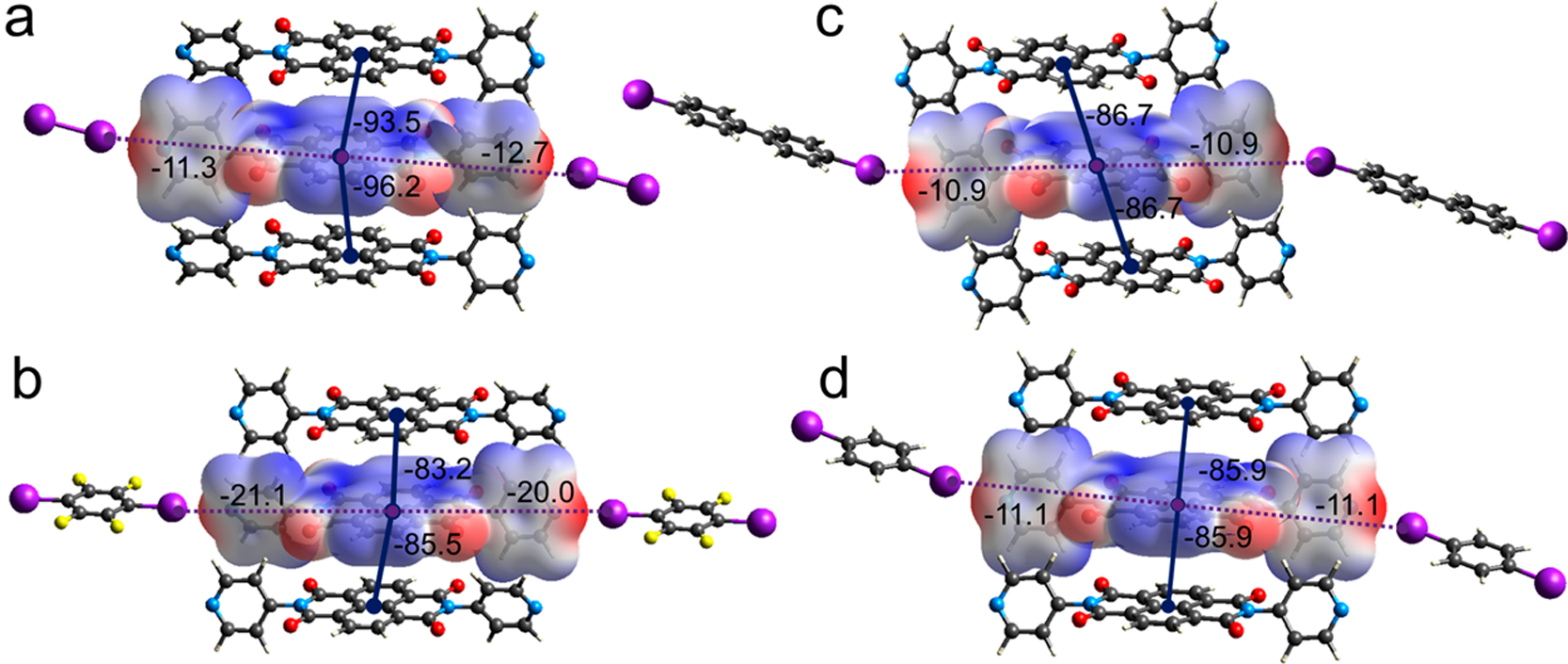
Simplified energy framework in **1-I**_**2**_ (a), **1-DITFB** (b), **1-DIBPH** (c), **1-DIB** (d). Molecular electrostatic potential (MEP) plotted on the electron density surface (drawn at the 0.002 au level) for **1** in all cocrystals. Blue lines represent the dispersive contribution to the energy framework, while dashed purple lines represent the halogen bonds. Stabilizing contributions are reported in kJ/mol.

Estimation of the interaction energy and energy framework was performed with CrystalExplorer17 and correlated with the Aromatic Analyzer tool in CSD Materials from the CCDC. Because of the presence of the iodine atoms in the cocrystal structures, all wave functions were calculated at the HF/3-21G level of theory.^37^ The interactions between piled NDI molecules result as the most stabilizing contribution ranging from −83.2/-85.5 kJ/mol in **1-DITFB** to −93.5/–96.2 kJ/mol in **1-I**_**2**_ (Figure 5). It is worth noting that these interactions are dominated by dispersive and Coulombic contributions between NDI molecules (Figures S29–S32, Tables S1–S8).

**Figure 6:**
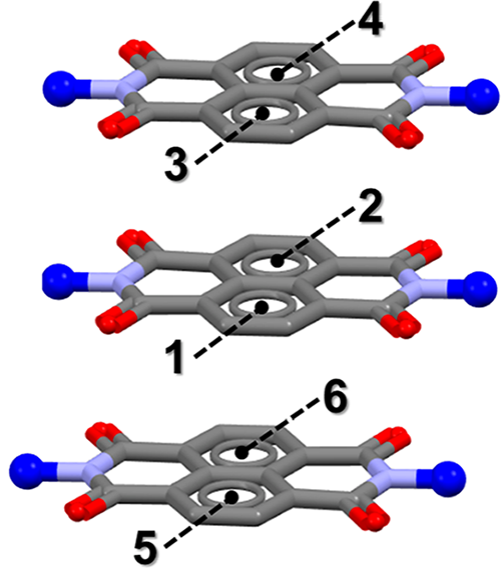
Schematic representation of interacting NDI molecules motif observed in all cocrystal structures. Labels indicate the number of the aromatic rings in the naphthalene cores consistent with results reported in Table 3. Atoms are colored according to the color code: C = gray, O = red, *N* = light blue. Terminal blue balls represent the pyridine rings in the NDI molecules.

The naphthalene core of each NDI molecules is not equally involved in π–π contact. In Figure 6, we report a schematic representation of three NDI molecules piled as a repeating motif observed in the cocrystal structures. Aromatic rings are individually labeled to better discriminate the main contribution of each ring to the dispersive interaction, also summarized inTable 3 (see Supporting Information for details). The naphthalene rings interact typically following an alternate pattern which is in agreement with the high roll angle and *D*_off_ reported above. Nevertheless, the lower roll angle and *D*_off_ observed for **1-I**_**2**_ are consistent with the exception of the alternate interaction path observed between naphthalene rings (Table 3). Whereas the XB angles in **1-I**_**2**_ and **1-DITFB** are quite similar, the energy associated with the halogen-bond interaction in the two cocrystals differs significantly (−21.1 and −11.3 kJ/mol respectively) (Figure 5). The aromatic ring of DITFB is electron poor due to the withdrawing effect of the fluorine substituents that strongly influences the σ-hole of the iodine atoms, thus enhancing the halogen-bond strength.

**Table 2:** Crystallographic Data and Structure Refinement

identification code	**1-DIB**	**1-DITFB**	**1-DIBPH**	**1-I**_**2**_
empirical formula	C_30_H_16_I_2_N_4_O_4_	C_30_H_12_F_4_I_2_N_4_O_4_	C_36_H_20_I_2_N_4_O_4_	C_24_H_12_I_2_N_4_O_4_
formula weight	750.27	822.24	826.36	674.18
temp/K	293(2)	293(2)	293(2)	296.15
crystal system	triclinic	triclinic	triclinic	triclinic
space group	*P*1̅	*P*1̅	*P*1̅	*P*1̅
*a*/Å	5.3753(8)	9.8430(14)	5.3330(8)	9.2012(12)
*b*/Å	10.9675(16)	10.7528(15)	11.7660(18)	10.2240(13)
*c*/Å	11.5660(16)	13.5633(19)	13.344(2)	11.8318(15)
*α*/*°*	74.520(2)	83.888(2)	113.977(2)	78.084(2)
*β*/*°*	78.023(2)	85.711(2)	94.197(3)	84.420(2)
*γ*/*°*	83.208(2)	78.97	103.053(2)	85.457(2)
volume/Å^3^	641.39(16)	1398.8(3)	732.4(2)	1081.9(2)
*Z*	1	2	1	2
ρ_calc_, g*/*cm^3^	1.942	1.952	1.874	2.069
μ/mm^-1^	2.498	2.320	2.198	2.949
*F*(000)	362.0	788.0	402.0	644.0
crystal size/mm^3^	1.2 × 0.3 × 0.3	1.2 × 0.4 × 0.3	1.1 × 0.5 × 0.5	1.1 × 0.4 × 0.3
radiation	MoKα	MoKα	MoKα	MoKα
2Θ range for data collection/°	3.718–62.274	3.024–63.786	3.4–57.234	3.53–60.652
index ranges	–7 ≤ *h* ≤ 7	–14 ≤ *h* ≤ 14	–7 ≤ *h* ≤ 7	–13 ≤ *h* ≤ 13
	–15 ≤ *k* ≤ 15	–15 ≤ *k* ≤ 15	–15 ≤ *k* ≤ 15	–14 ≤ *k* ≤ 14
	–16 ≤ *l* ≤ 16	–20 ≤ *l* ≤ 20	–17 ≤ *l* ≤ 17	–16 ≤ *l* ≤ 16
reflections collected	10482	23248	10570	17450
independent reflections	3984	8955	3727	6457
	*R*_int_ = 0.0353	*R*_int_ = 0.0659	*R*_int_ = 0.0419	*R*_int_ = 0.0505
	*R*_sigma_ = 0.0443	*R*_sigma_ = 0.0917	*R*_sigma_ = 0.0497	*R*_sigma_ = 0.0643
data restraints parameters	3984	8955	3727	6457
	0	0	0	0
	181	397	248	307
goodness-of-fit on *F*^2^	1.035	0.955	0.982	1.004
final *R* indexes [*I* ≥ 2σ(*I*)]	*R*_1_ = 0.0374, *wR*_2_ = 0.0766	*R*_1_ = 0.0466, *wR*_2_ = 0.0872	*R*_1_ = 0.0371, *wR*_2_ = 0.0756	*R*_1_ = 0.0399, *wR*_2_ = 0.0793
final *R* indexes [all data]	*R*_1_ = 0.0613, *wR*_2_ = 0.0859	*R*_1_ = 0.1163, *wR*_2_ = 0.1085	*R*_1_ = 0.0629, *wR*_2_ = 0.0848	*R*_1_ = 0.0826, *wR*_2_ = 0.0958
largest diff. peak/hole/e·Å^*–*3^	0.86/–0.88	0.62/–0.59	0.73/–0.32	0.66/–0.64

**Table 3:** Aromatic Analyzer Results^a^

	aromatic ring					
cocrystal	#1	#2	distance (Å)	relative orientation (deg)	intermolecular score	aromatic analyzer assessment
**1-I_2_**	2	3	3.59	0	8.8	strong
	2	6	4.56	1.39	8.3	strong
**1-DIB**	1	4	3.86	0	10	strong
	2	5	3.86	0	10	strong
**1-DIBPH**	1	4	3.87	0	10	strong
	2	5	3.87	0	10	strong
**1-DITFB**	1	4	3.56	0	9.2	strong
	2	5	3.52	0	8.4	strong

a Aromatic rings are labeled according to Figure 6.

## Conclusions

We have clearly demonstrated that the poorly soluble *N*,*N*′-di(4-pyridyl)-naphthalenediimmide (**1**) can be successfully used to make XB-based cocrystals with four different iodo-containing XB donors. In particular, the mechanochemical protocol adopted has allowed to easily obtain the target products, which are otherwise hard to synthesize due to the low solubility of **1**. An in-depth structural analysis supported by in-silico investigation has evidenced that the solid frameworks of the four new compounds are featured by strong π–π interactions between piled molecules of **1**, which result in the most stabilizing contributions dominated by dispersive and Coulombic interactions, ranging from −83.2/–85.5 kJ/mol in **1-DITFB** to −93.5/–96.2 kJ/mol in **1-I**_**2**_, largely exceeding the XB contributions. The pyridine moieties have shown a remarkable ability as halogen-bond acceptors returning similar patterns, where 1D chains develop along the halogen-bond direction.

## Supporting Information Available

ORTEP figures, intermolecular interactions analyses, PXRD, TGA, NMR, IR, EI-MS, and energy frameworks (PDF)

## Accession Codes

CCDC 2079372–2079375 contain the supplementary crystallographic data for this paper. These data can be obtained free of charge via www.ccdc.cam.ac.uk/data_request/cif, or by emailing data_request@ccdc.cam.ac.uk, or by contacting The Cambridge Crystallographic Data Centre, 12 Union Road, Cambridge CB2 1EZ, UK; fax: +44 1223 336033.

## Acronyms

**1**: *N*,*N*′-di(4-pyridyl)-naphthalene-1,4,5,8-tetracarboxydiimide
DIB: 1,4-diiodobenzene
DIBPH: 4,4′-diiodobiphenylene
DITFB: 1,4-diiodotetrafluorobenzene
DMF: *N*,*N*-dimethylformamide
DMSO: dimethylsolfoxide
HB: hydrogen bond
I_2_: molecular iodine
TFA: tetrafluoroacetic acid
XB: halogen bond
