# Supplementary material for: Mechanochemical Preparation of Dipyridyl-Naphthalenediimide Cocrystals: Relative Role of Halogen-Bond and π–π Interactions
Source: Cryst Growth Des. 2021 Sep 1;21(10):5687–96. doi: 10.1021/acs.cgd.1c00531 (PMC8498987; doi:10.1021/acs.cgd.1c00531)
Supplement: Supplementary file 1 — cg1c00531_si_001.pdf [file cg1c00531_si_001.pdf]

Mechanochemical preparation of dipyridyl-naphthalenediimide cocrystals: the relative role of halogen-bond and π-π interactions

Paolo Pio Mazzeo[a,b], Marianna Pioli[a], Fabio Montisci[a], Alessia Bacchi*[a,b], Paolo Pelagatti*[a,c]

a Dipartimento di Scienze Chimiche, della Vita e della Sostenibilità Ambientale, Università di Parma, Parco Area delle Scienze 17/A, 43124 Parma, Italy, alessia.bacchi@unipr.it, paolo.pelagatti@unipr.it
b Biopharmanet-TEC, Università di Parma, Parco Area delle Scienze 27/A, 43124 Parma, Italy
c Centro Interuniversitario di Reattività Chimica e Catalisi (CIRCC), Via Celso Ulpiani 27, 70126 Bari, Italy

Supporting Information

General 2
Crystallography 3
Analysis of Intermolecular aromatic interactions 5
Additional Statistical Analysis 7
X-ray Powder Diffraction - XRPD 8
Thermal Gravimetric Analysis – TGA 10
Nuclear Magnetic Resonance – ^{1}H-NMR 12
Infrared Spectroscopy 16
EI-MS Spectrometry 16
Energy Frameworks calculation 19
Full interaction map 27
Geometrical description 27

General
Photographs of the crystals of 1-DIB and 1-DIBPH

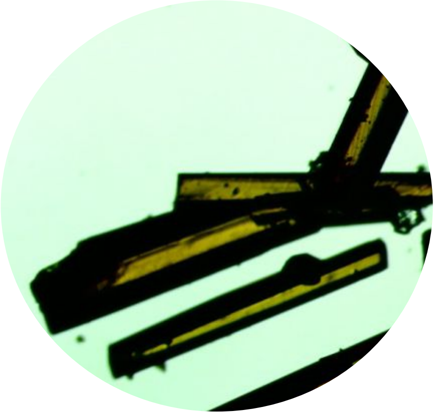

Figure S1: Orange needles crystals of **1·DIB**.

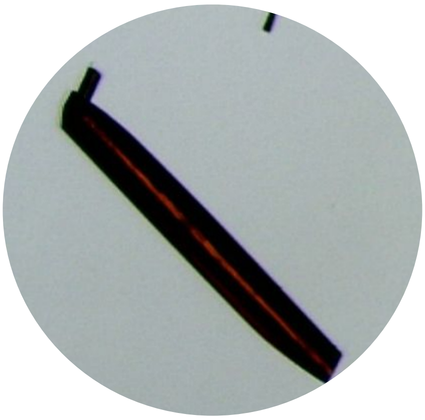

Figure S2: Orange needles crystals of **1-DIBPH**.

Crystallography

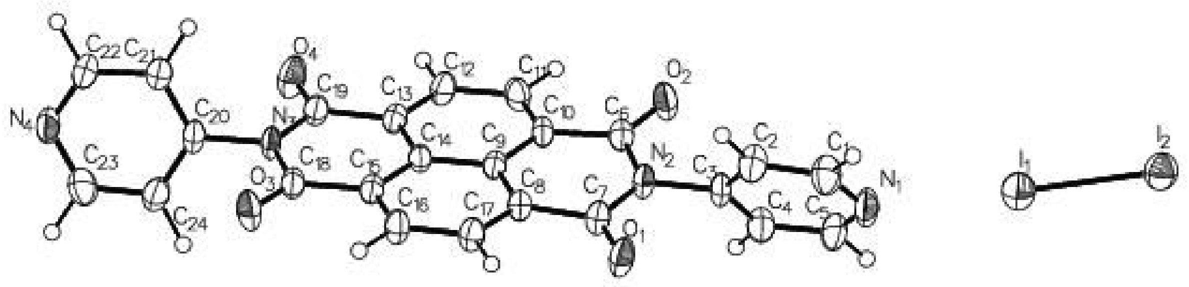

Figure S3: ORTEP drawing of **1-I$_2$** with labelling and thermal ellipsoids for non-H atoms at the 50% probability level.

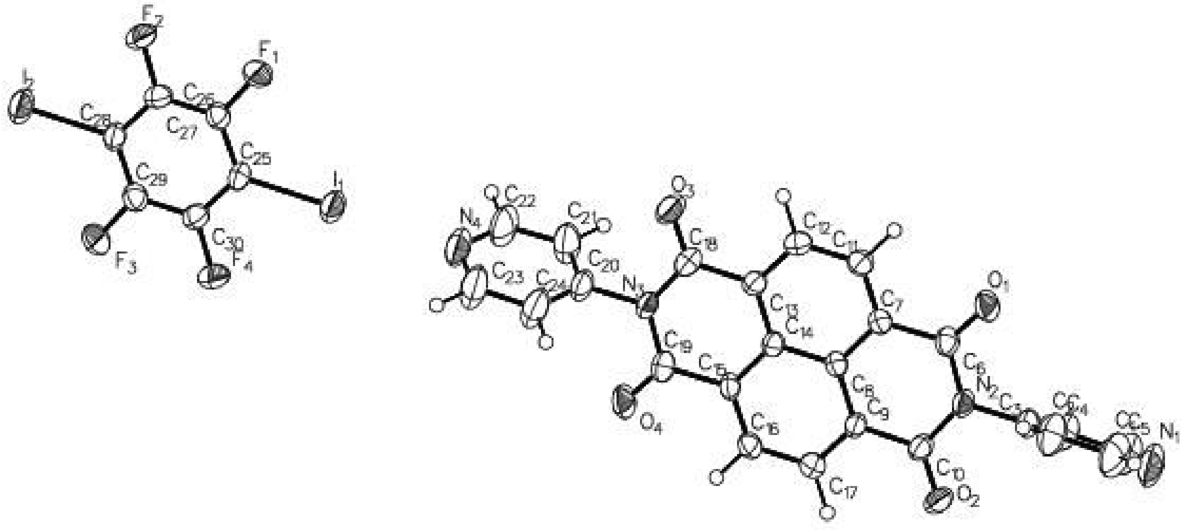

Figure S4: ORTEP drawing of **1-DITFB** with labelling and thermal ellipsoids for non-H atoms at the 50% probability level.

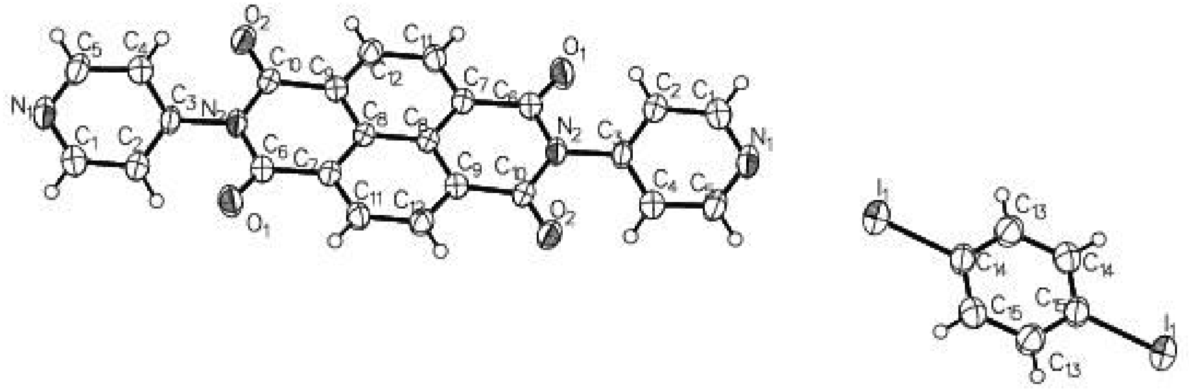

Figure S5: ORTEP drawing of **1-DIB** with labelling and thermal ellipsoids for non-H atoms at the 50% probability level.

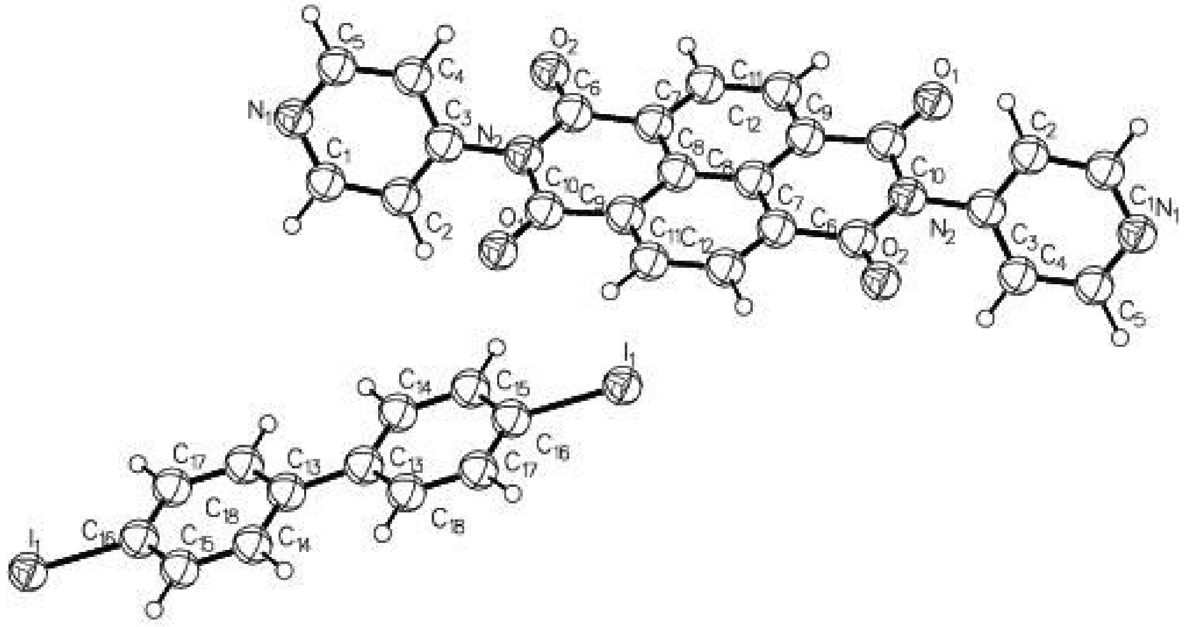

Figure S6: ORTEP drawing of **1-DIBPH** with labelling and thermal ellipsoids for non-H atoms at the 50% probability level.

Analysis of Intermolecular aromatic interactions

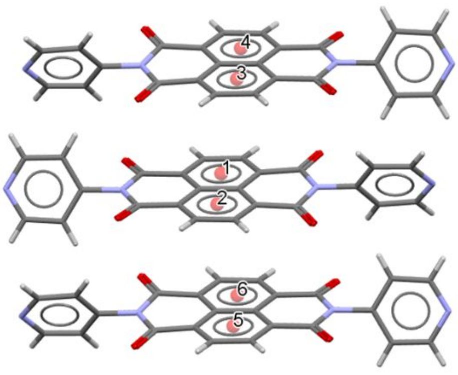

Centroid #1	Centroid #2	Distance (Å)	Relative Orientation (°)	Intermolecular Score	Assessment
1	3	3.59	0	8.8	Strong
1	6	4.56	1.39	8.3	Strong
2	6	3.53	0	7.9	Strong
1	4	4.73	1.39	7.7	Strong
1	5	6.4	0	3.4	Moderate
2	4	6.61	0	2.9	Weak

Figure S7: Analysis of intermolecular aromatic interaction for NDI π-π stacking in **1-I$_2$** according to CSD Material suite. Labelled centroids refer to aromatic rings of the naphthalene skeleton.

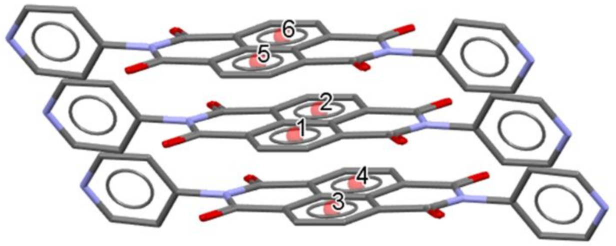

Centroid #1	Centroid #2	Distance (Å)	Relative Orientation (°)	Intermolecular Score	Assessment
1	6	3.86	0	10	Strong
2	3	3.86	0	10	Strong
1	3	5.38	0	6.1	Moderate
1	5	5.38	0	6.1	Moderate
2	4	5.39	0	6.1	Moderate
2	6	7.4	0	1.6	Weak
2	5	7.4	0	1.6	Weak

Figure S8: Analysis of intermolecular aromatic interaction for NDI π-π stacking in **1-DIB** according to CSD Material suite. Labelled centroids refer to aromatic rings of the naphthalene skeleton. Hydrogen atoms are removed for the sake of clarity.

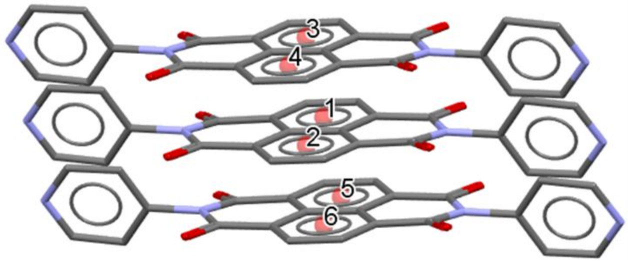

Centroid #1	Centroid #2	Distance (Å)	Relative Orientation (°)	Intermolecular Score	Assessment
1	6	3.87	0	10	Strong
2	3	3.87	0	10	Strong
1	3	5.33	0	6.1	Moderate
1	5	5.33	0	6.1	Moderate
2	4	5.33	0	6.1	Moderate
2	6	5.33	0	6.1	Weak
1	4	7.33	0	1.7	Weak
2	5	7.4	0	1.6	Weak

Figure S9: Analysis of intermolecular aromatic interaction for NDI π-π stacking in **1-DIBPH** according to CSD Material suite. Labelled centroids refer to aromatic rings of the naphthalene skeleton. Hydrogen atoms are removed for the sake of clarity.

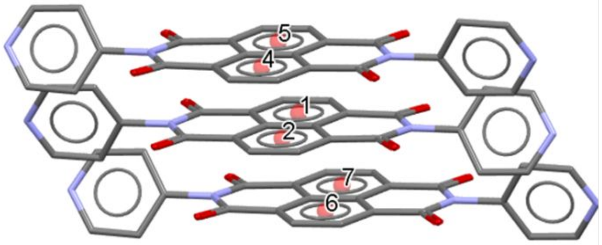

Centroid #1	Centroid #2	Distance (Å)	Relative Orientation (°)	Intermolecular Score	Assessment
2	5	3.56	0	9.2	Strong
1	6	3.52	0	8.4	Strong
1	7	4.89	0.43	7.9	Strong
2	6	4.89	0.43	7.9	Strong
1	5	4.97	0.43	7.7	Strong
2	4	4.97	0.43	7.7	Strong
2	7	6.88	0	2.4	Weak
1	4	6.98	0	2.3	Weak

Figure S10: Analysis of intermolecular aromatic interaction for NDI π-π stacking in **1-DITFB** according to CSD Material suite. Labelled centroids refer to aromatic rings of the naphthalene skeleton. Hydrogen atoms are removed for the sake of clarity.

Additional Statistical Analysis

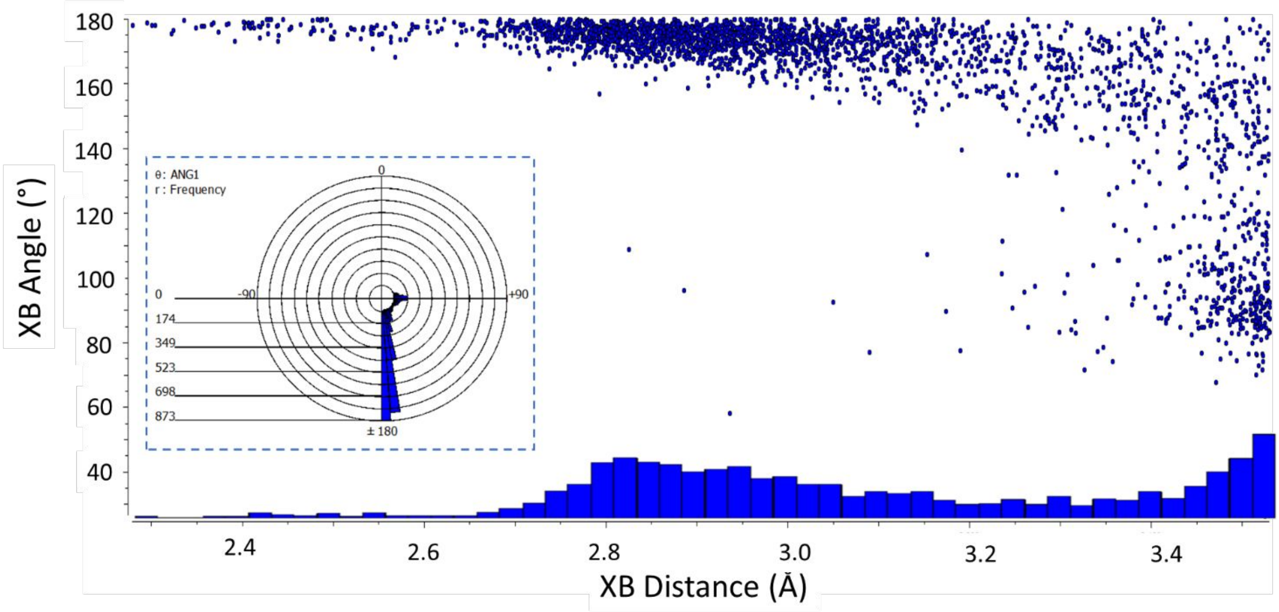

Figure S11: Scatter plot showing the dependence of halogen bond (XB) angles as a function of the halogen bond distance in the CCDC CSD 5.42 for the query N---I-X, (X = any atom) with the iodine atoms as terminal (coordination number = 1). Total number of hits 2061. Inset: polar plot of the XB angle. Histogram reporting the XB distance distribution is also reported.

X-ray Powder Diffraction - XRPD

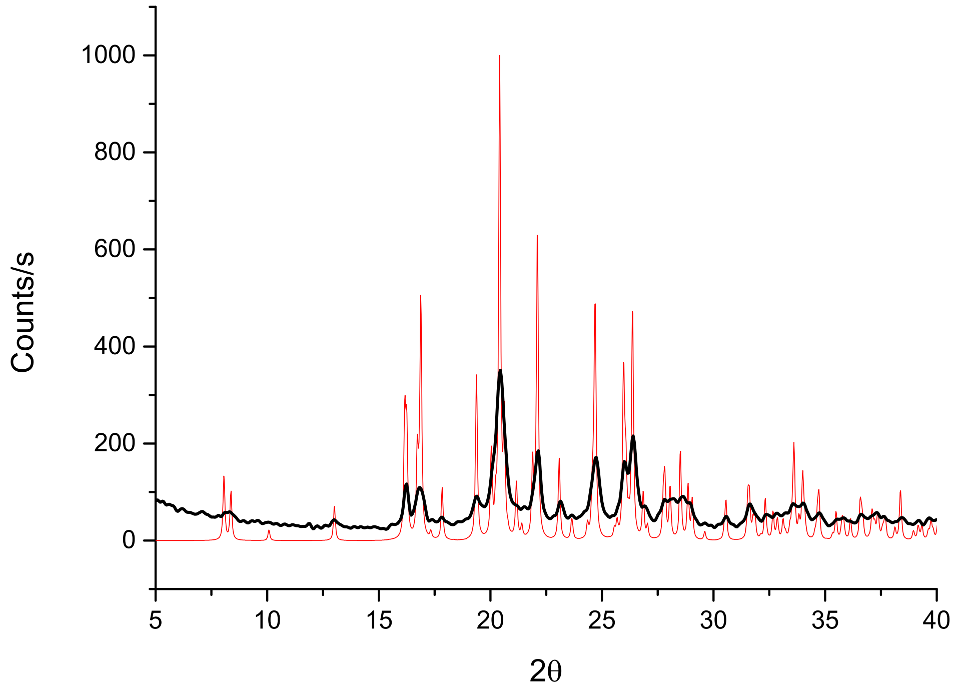

Figure S12: Comparison of experimental (black line) and calculated (red line) X-ray Powder Pattern of **1-DIB.**

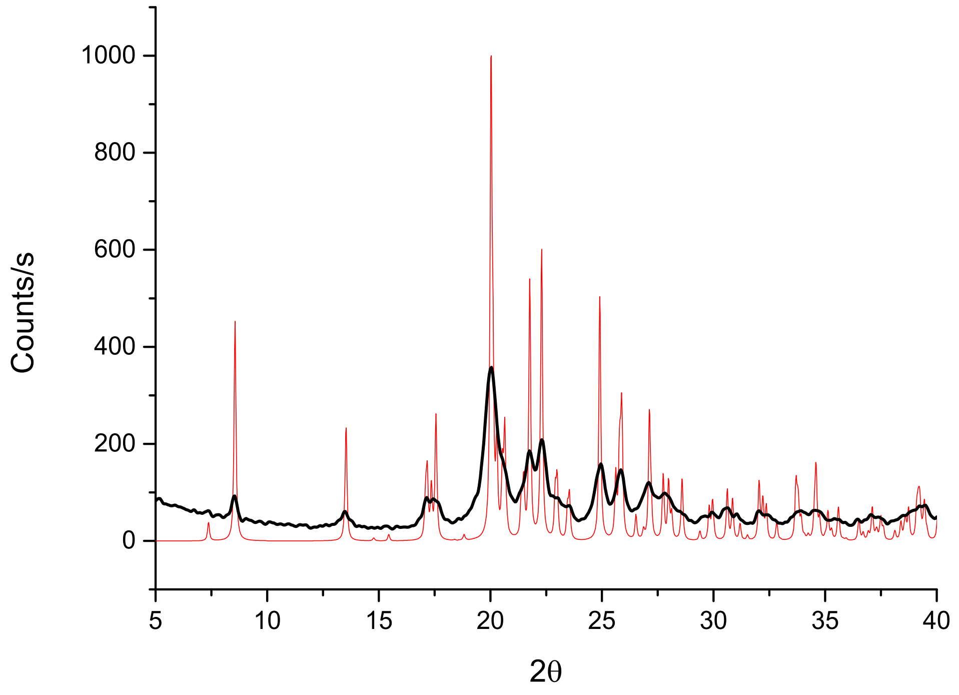

Figure S13: Comparison of experimental (black line) and calculated (red line) X-ray Powder Pattern of **1-DIBPH.**

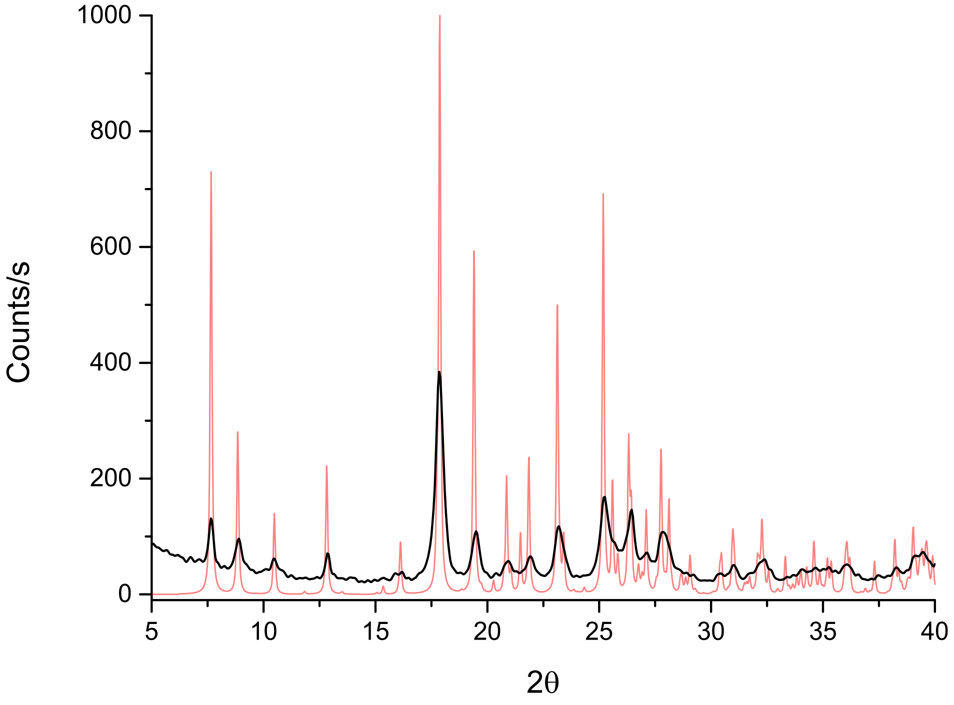

Figure S14: Comparison of experimental (black line) and calculated (red line) X-ray Powder Pattern of **1-I$_2$.**

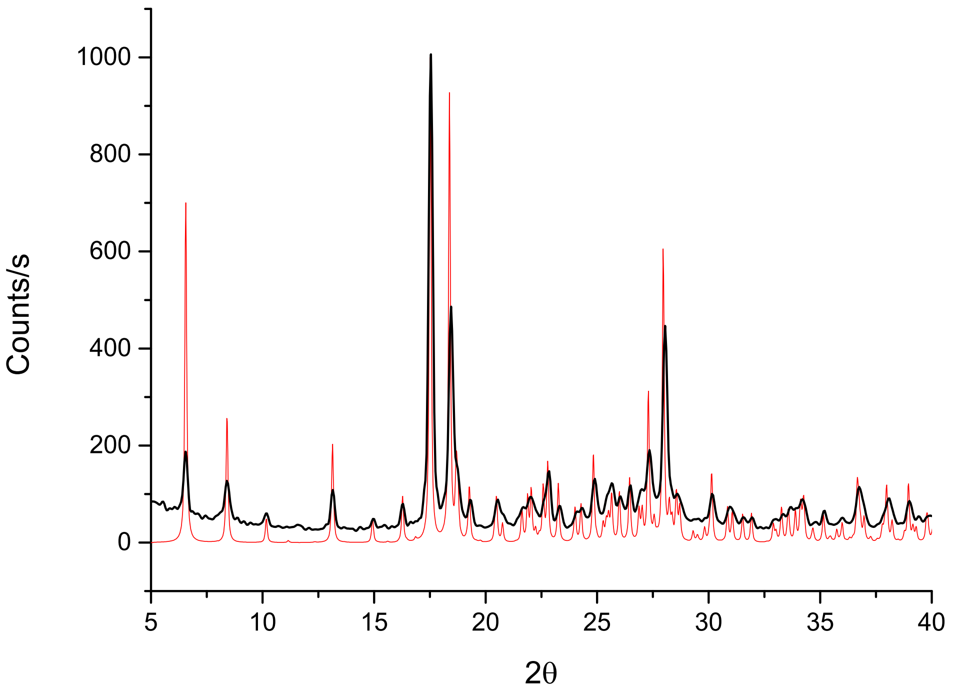

Figure S15: Comparison of experimental (black line) and calculated (red line) X-ray Powder Pattern of **1-DITFB**.

Thermal Gravimetric Analysis – TGA

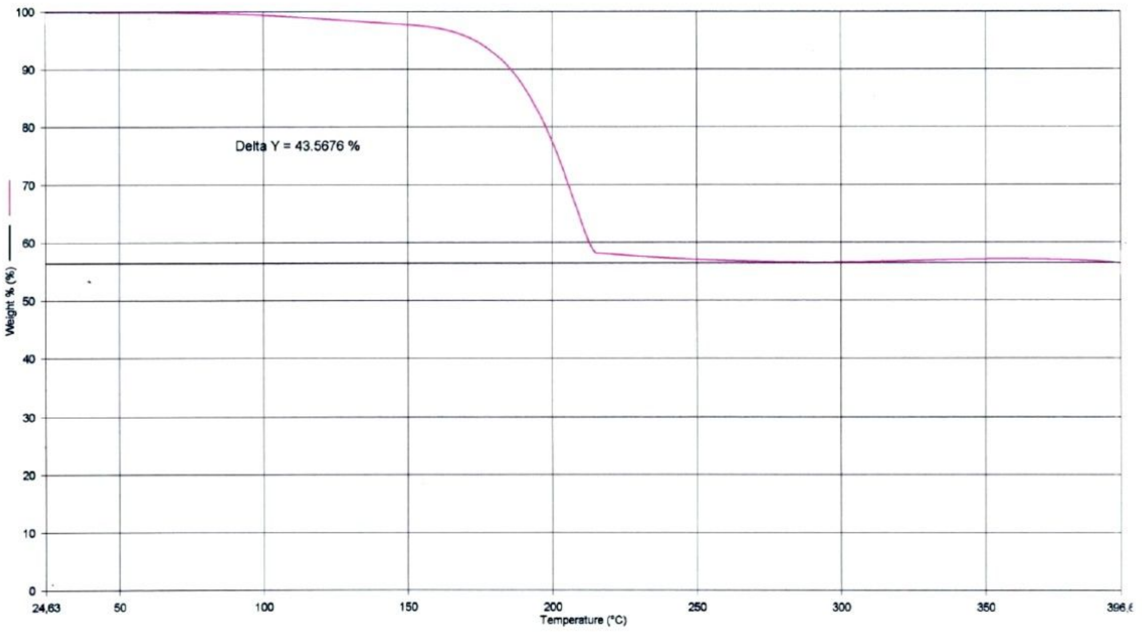

Figure S16: TGA plot of **1-DIB** from 30°C to 400°C at a scan rate of 10°/min

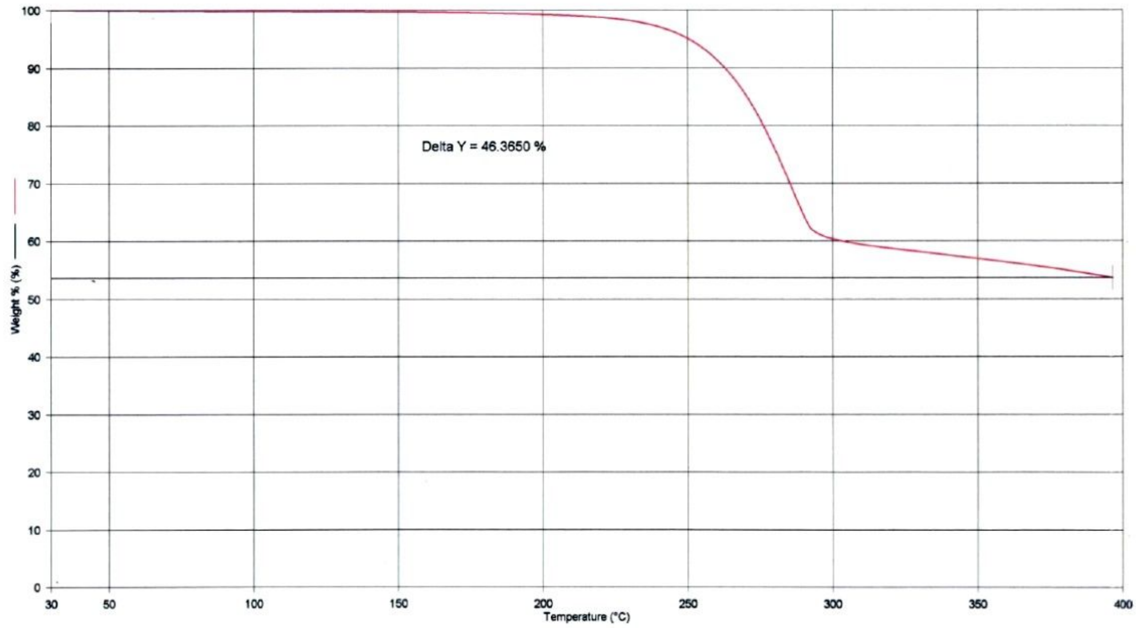

Figure S17: TGA plot of **1-DIBPH** from 30°C to 400°C at a scan rate of 10°/min

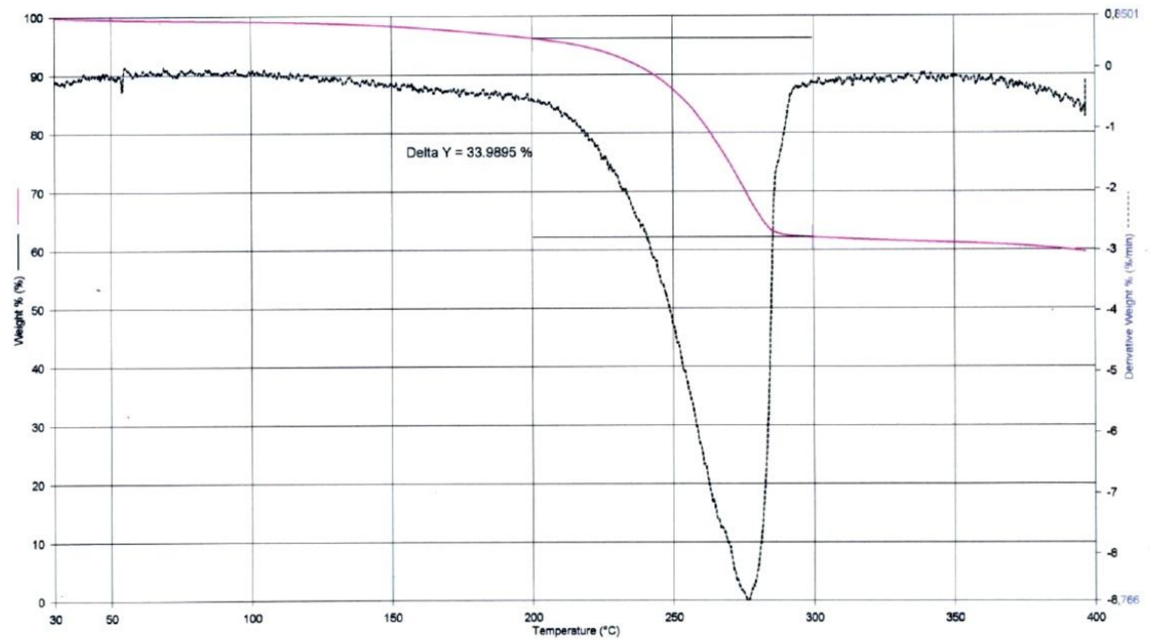

Figure S18: TGA plot of **1-I_2** from 30°C to 400°C at a scan rate of 10°/min

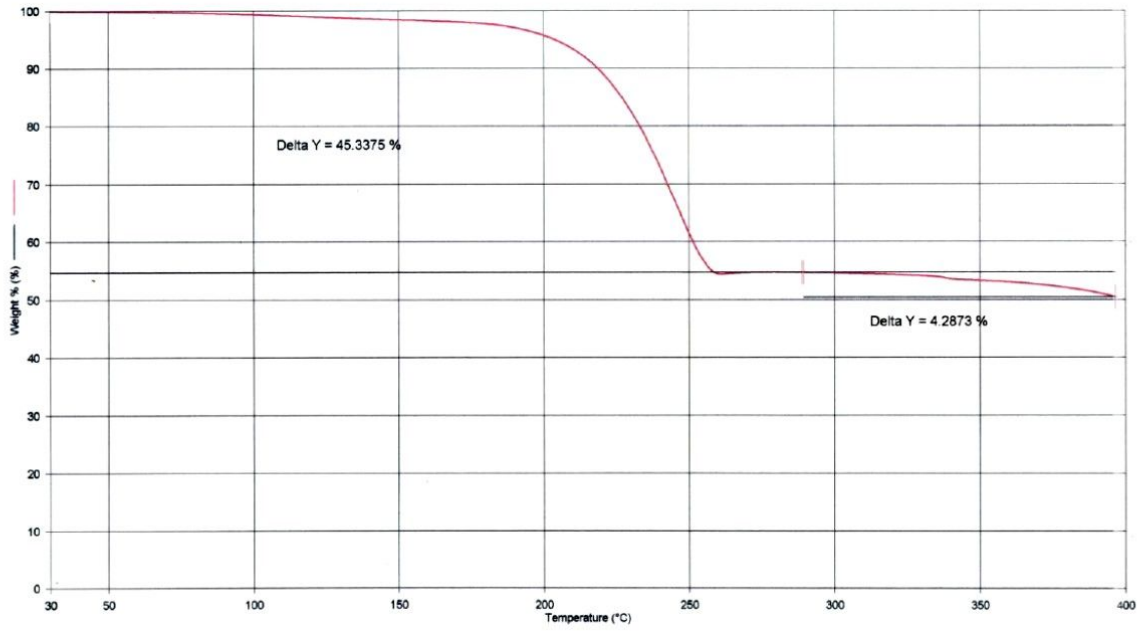

Figure S19: TGA plot of **1-DITFB** from 30°C to 400°C at a scan rate of 10°/min

Nuclear Magnetic Resonance – ^{1}H-NMR

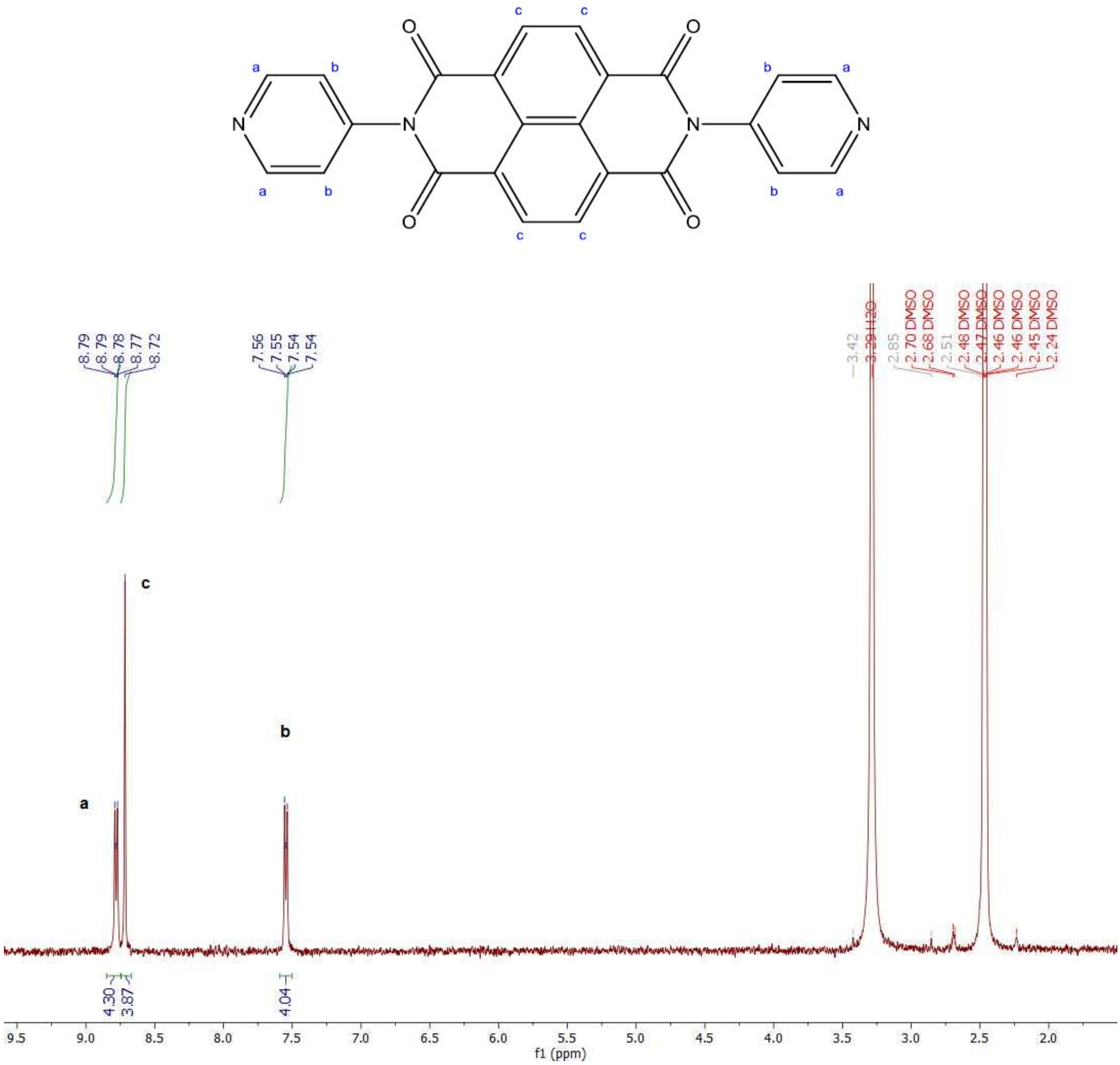

Figure S20: ^{1}H-MR of **1** in DMSO-d_6. Peaks are labelled accordingly to the chemical sketch reported.

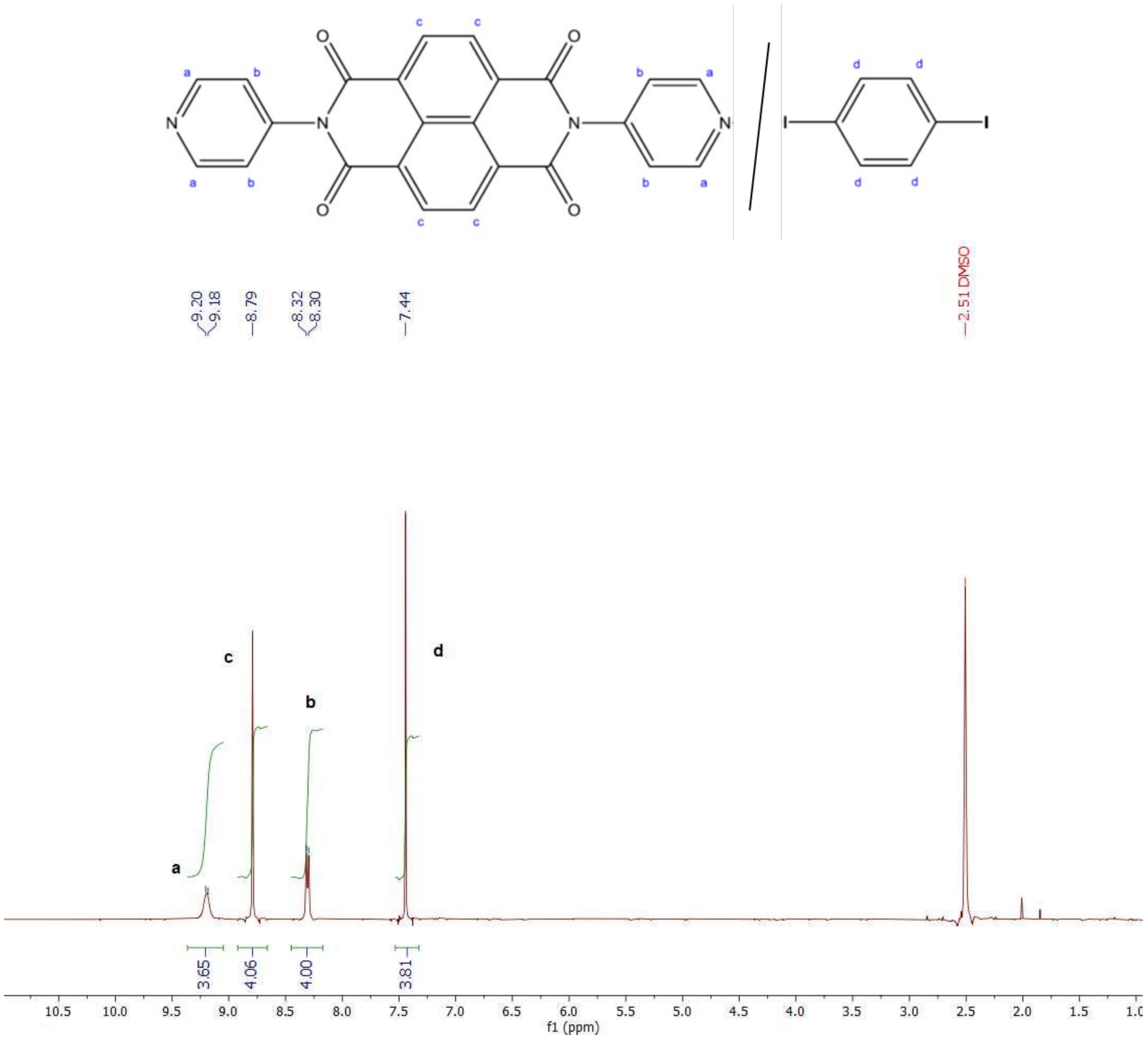

Figure S21: a) ^{1}H-NMR of **1-DIB** in DMSO-d_6/CF_3COOD. Peaks are labelled accordingly to the chemical sketch reported as insert. The signal labelled as d belong to the four protons of DIB.

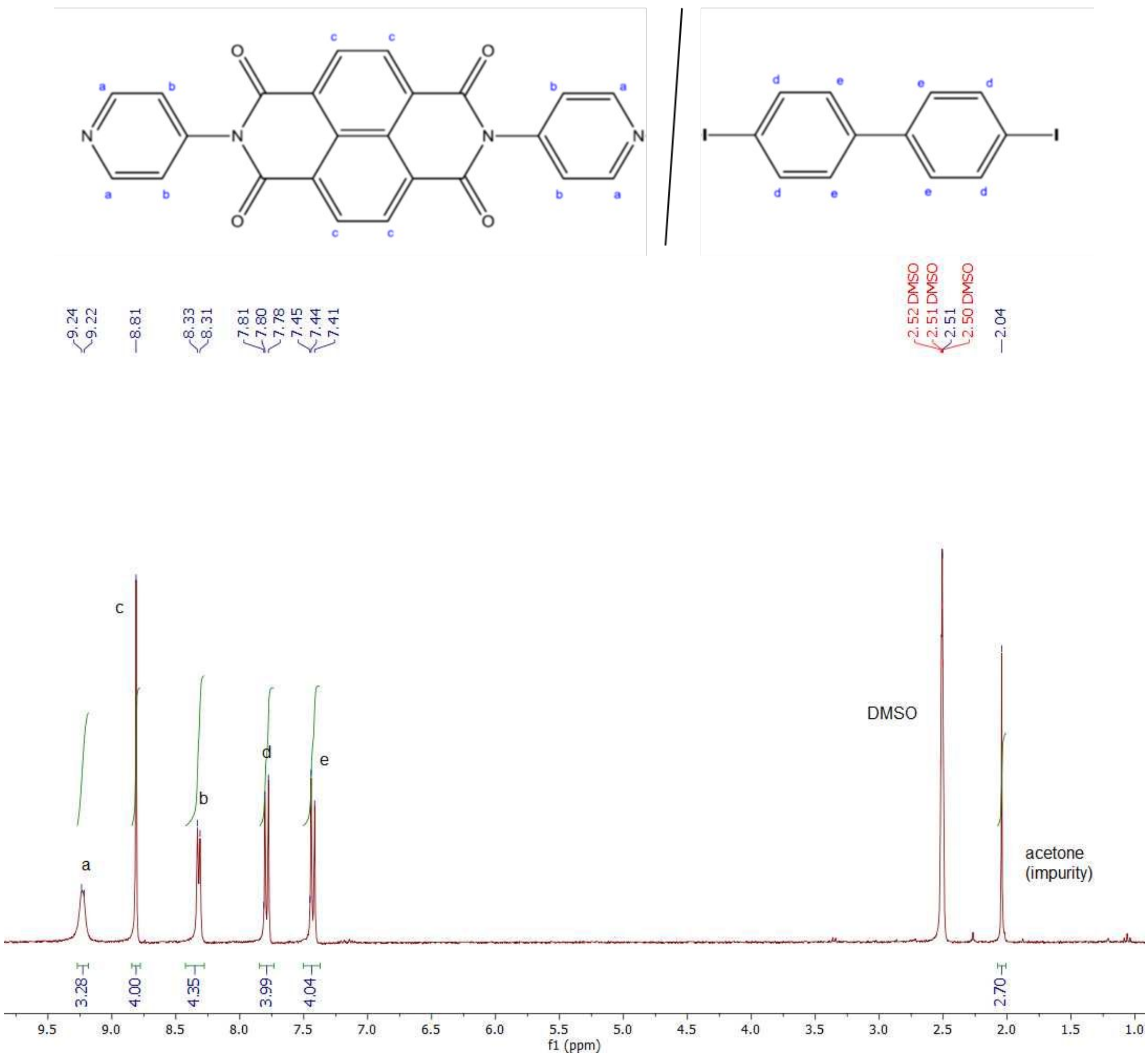

Figure S22: ^{1}H-NMR of **1-DIBPH** in DMSO-d_6/CF_3COOD. Peaks are labelled accordingly to the chemical sketch reported as insert. The signals labelled as d and e belong to DIBPH.

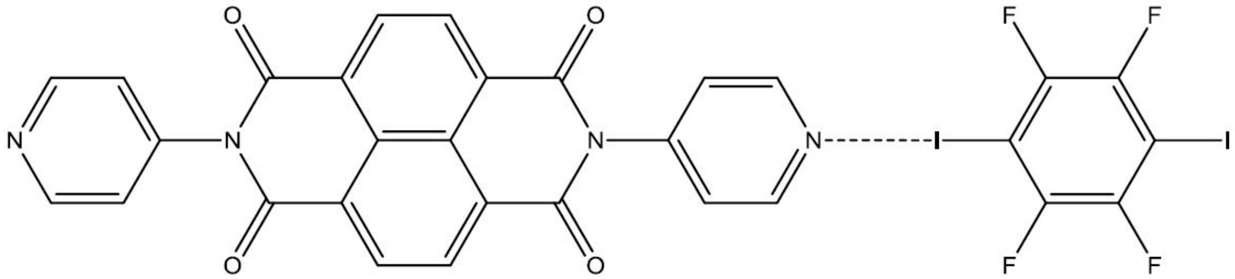

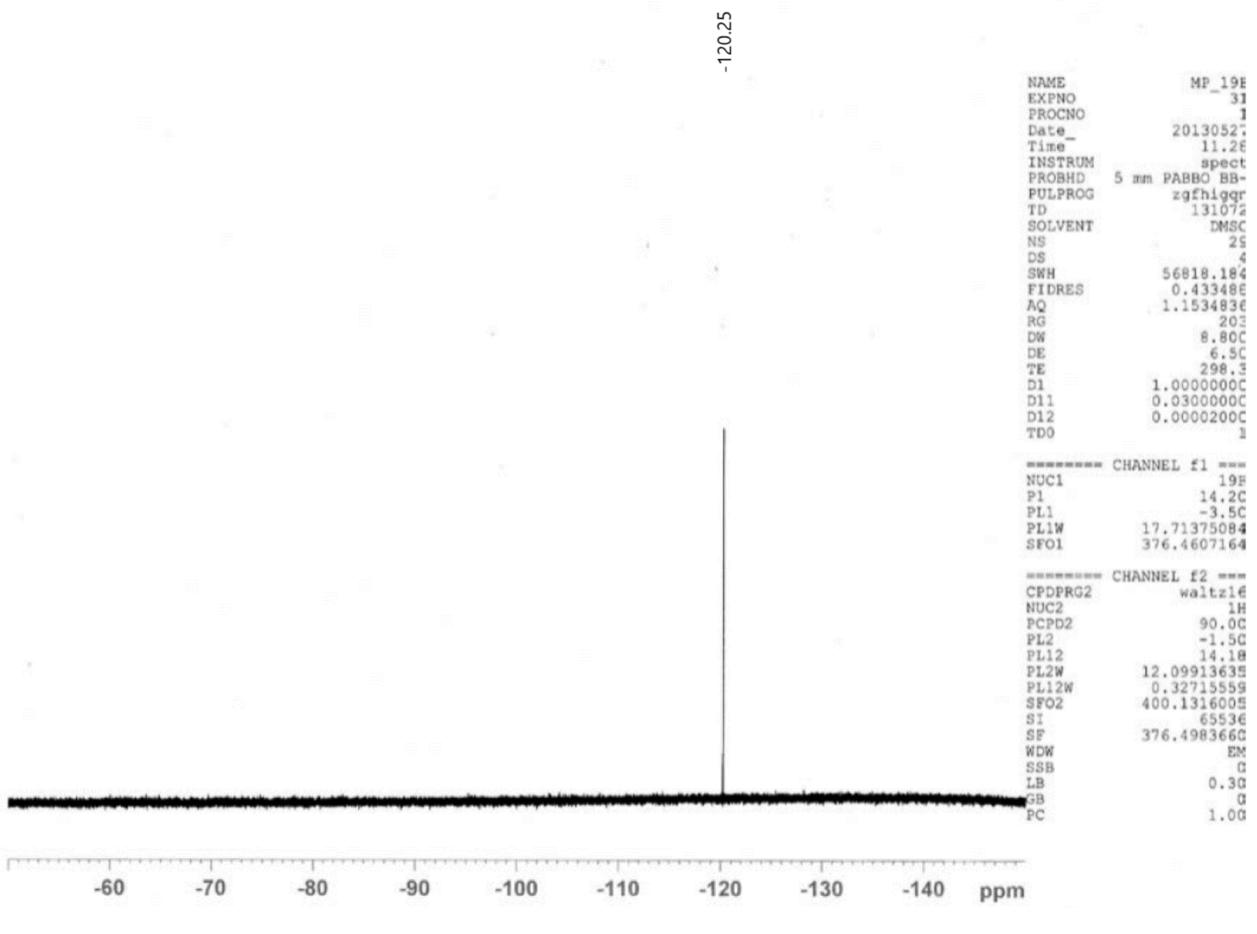

Figure S23: $^{19}F\{^{1}H\}$-NMR of **1-DITFB** in DMSO-d_6.

Infrared Spectroscopy

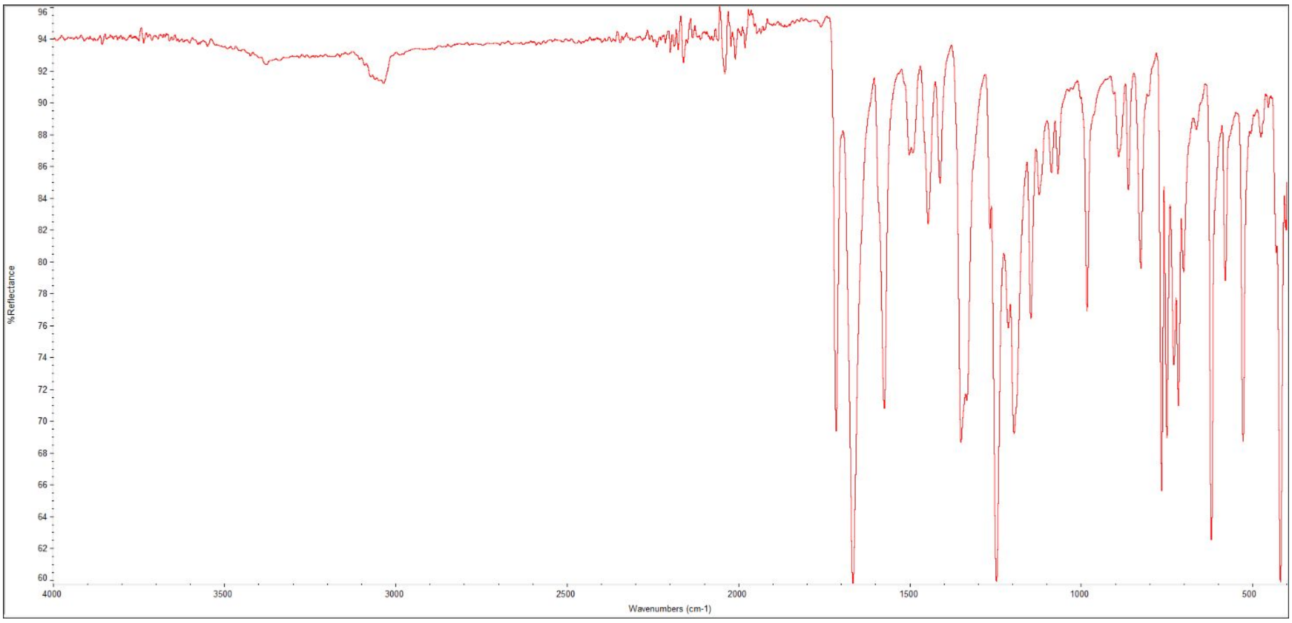

Figure S24 FTIR spectrum of **1** used to identify the precipitate initially formed during cooling of the reactant solutions. Main bands attribution (cm^{-1}): 3031, ν(C-H)$_{ar}$; 1715, ν(C=O), 1667, ν(C=O); 1575, ν(C=C)$_{ar}$

EI-MS Spectrometry

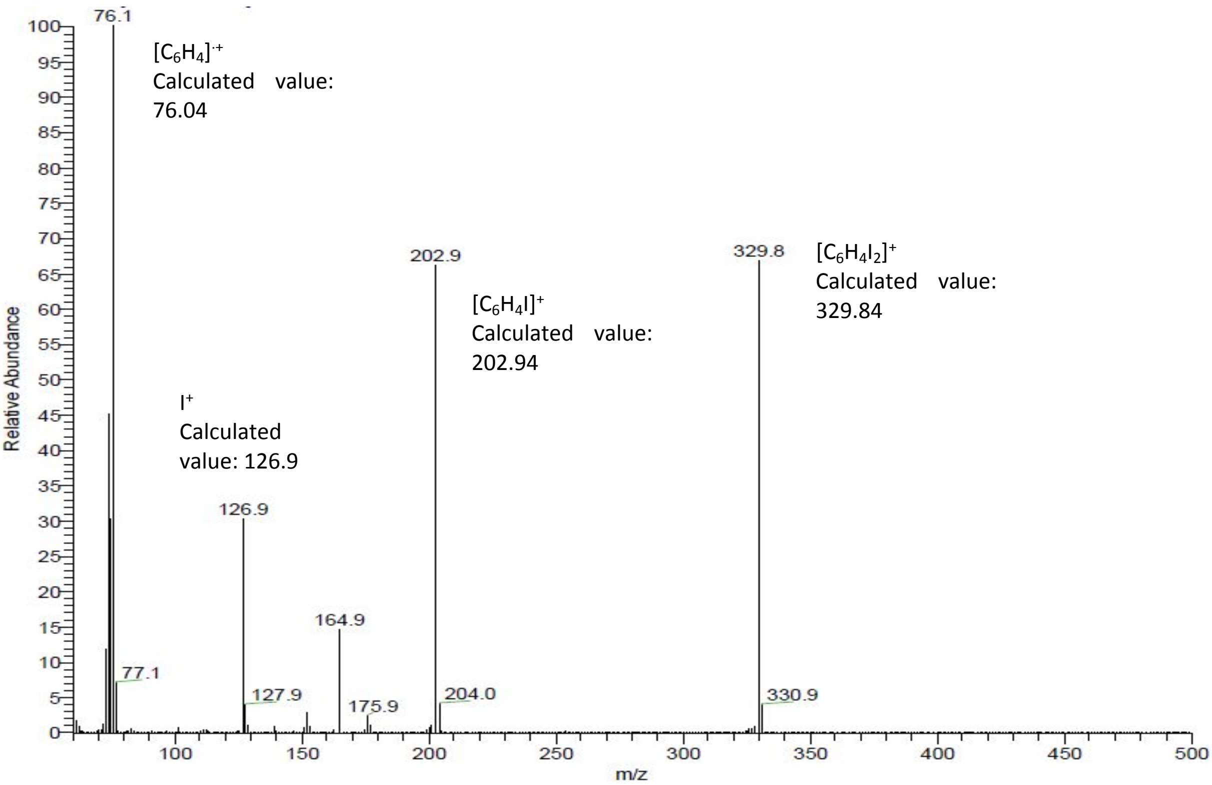

Figure S24: EI-MS(+) spectrum of **1-DIB**. Temperature probe: 150°C.

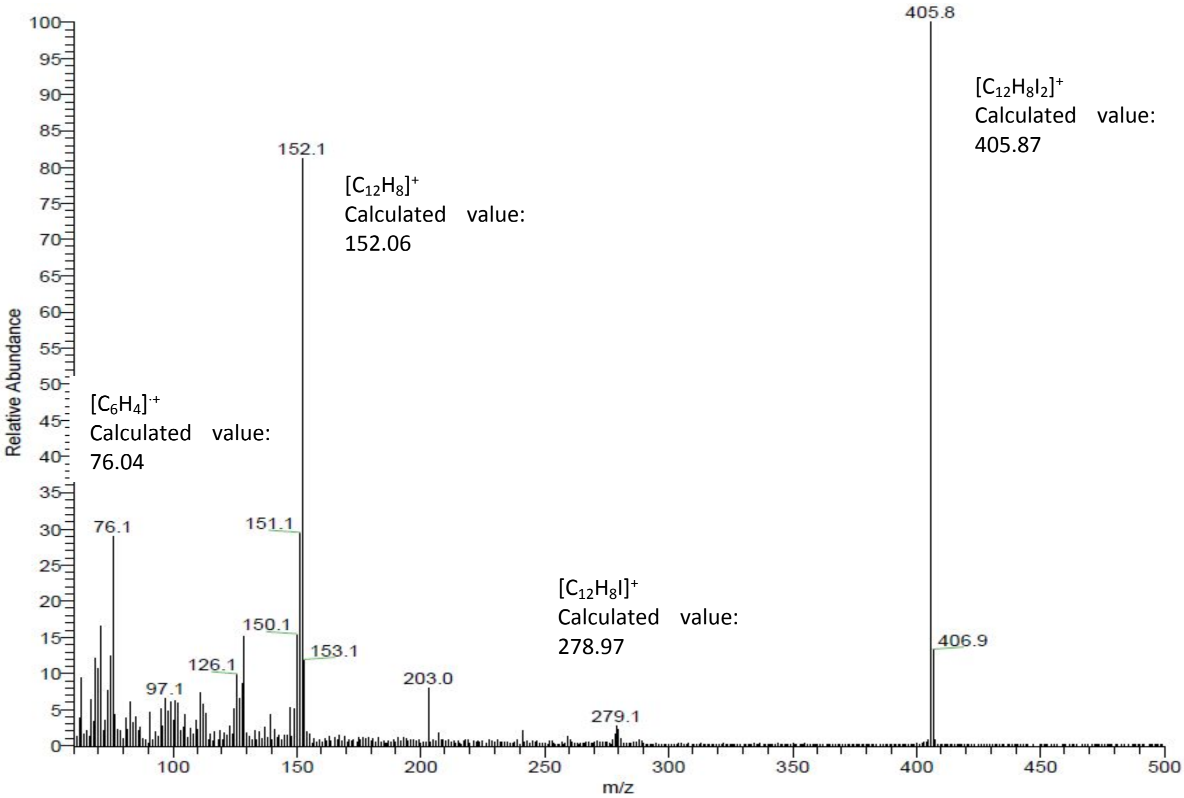

Figure S25: EI-MS(+) spectrum of **1-DIBPH**. Temperature probe: 150°C.

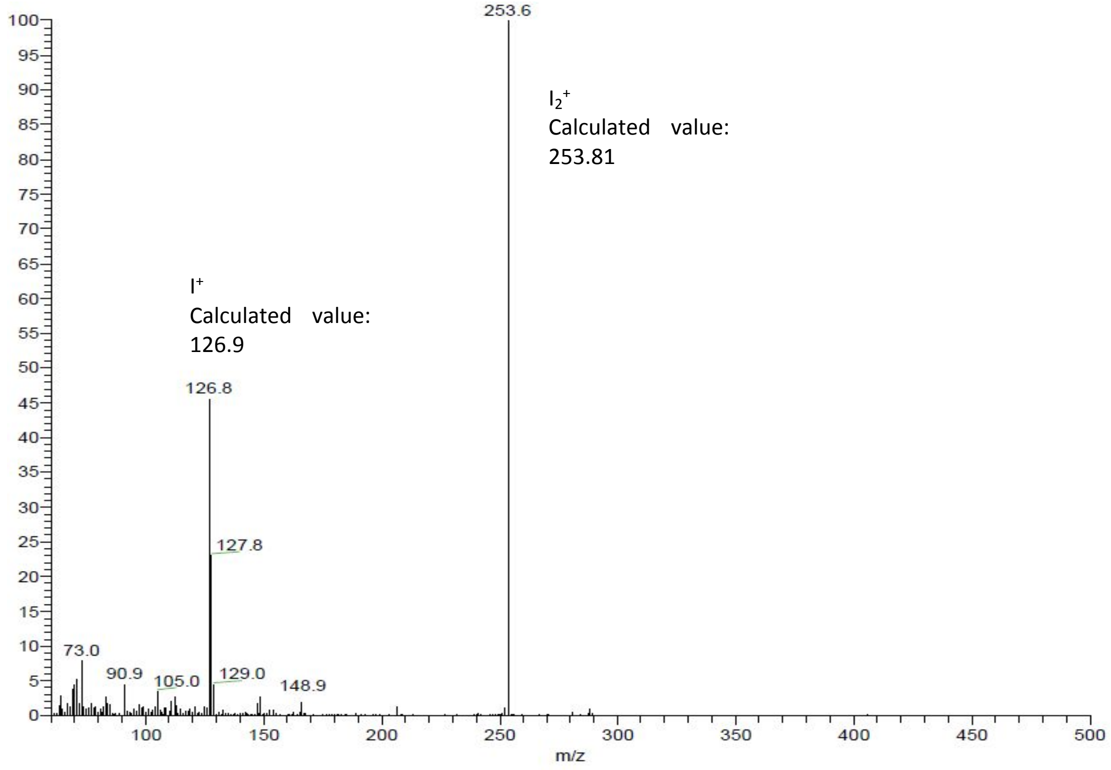

Figure S26: EI-MS(+) spectrum of **1-I$_2$**. Temperature probe: 150°C.

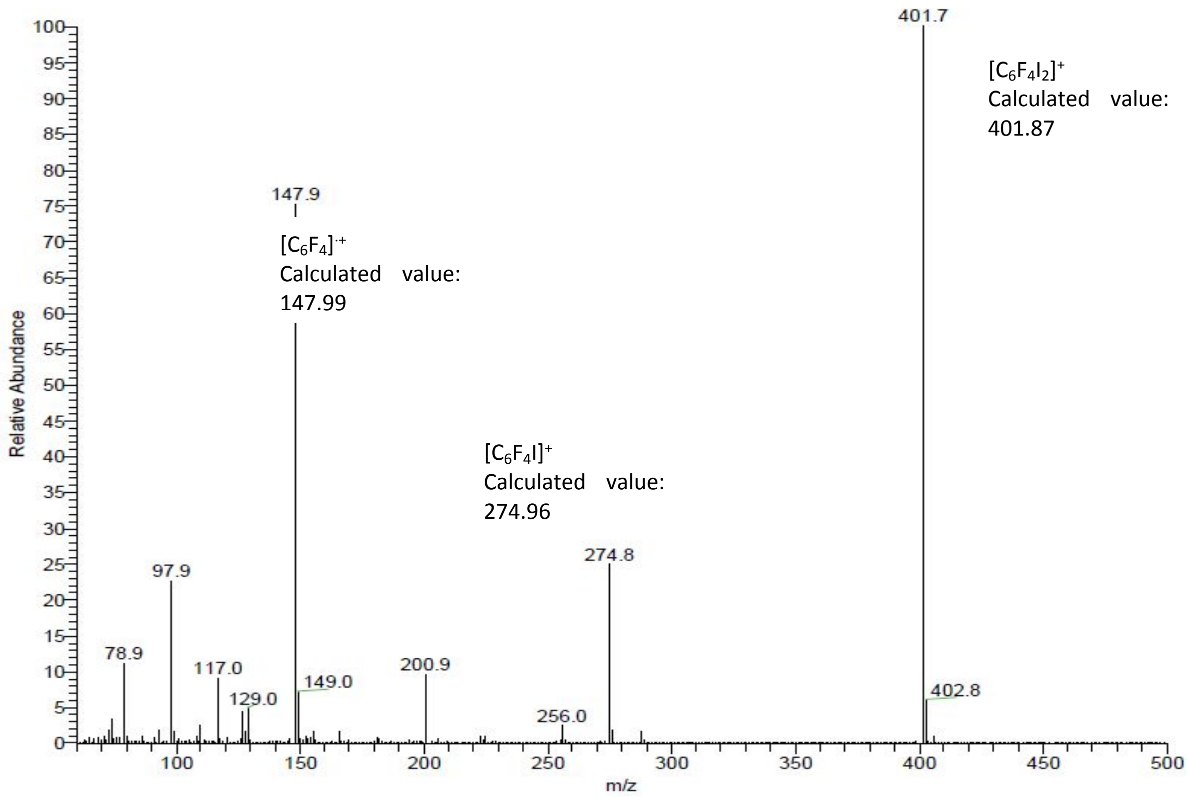

Figure S27: EI-MS(+) spectrum of **1-DITFB**. Temperature probe: 150°C.

Energy Frameworks calculation

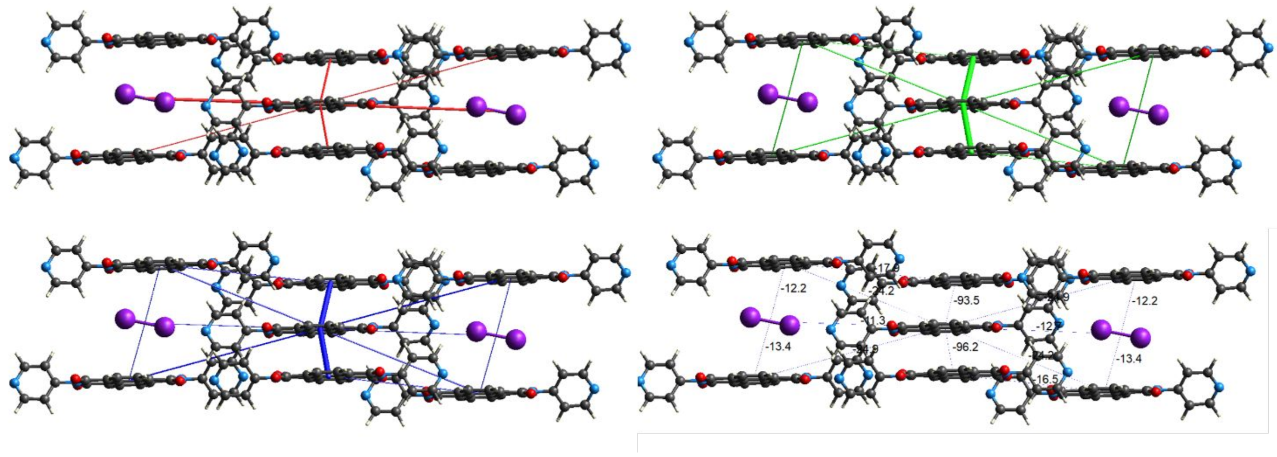

Figure S28: Energy decomposition of the main interactions for **1-I$_2$** (total=blue, dispersive=green, coulombic=red). Lines thickness are proportional to the intensity of the interactions (threshold =-10 kJ/mol). Annotated version with stabilizing interaction energy is reported in kJ/mol.

Table S1: Interaction Energies (kJ/mol) calculated for **1-I$_2$** within a radius of 5.0 Å from each atom in **1**. R is the distance between molecular centroids (mean atomic position) in Å. Total energies, reported for the benchmarked HF/3-21G level, are the sum of the four energy components, scaled appropriately (k_ele=1.019, k_pol=0.651, k_disp=0.901, k_rep= 0.811).

N	Sym. Op.	R	E_ele	E_pol	E_dis	E_rep	E_tot
1	-x, -y, -z	4.68	-33.4	-11.3	-109.4	57.2	-93.5
1	-x, -y, -z	12.56	1.1	-0.3	-2.7	0.0	-1.5
1	-x, -y, -z	15.13	-3.3	-0.6	-6.3	1.1	-8.4
2	x, y, z	11.83	-8.7	-3.0	-26.5	12.9	-24.2
1	-	6.55	-3.5	-1.2	-12.6	10.4	-7.3
1	-x, -y, -z	12.88	-7.8	-2.5	-11.2	2.2	-17.9
1	-x, -y, -z	4.62	-35.1	-11	-112.1	58.9	-96.2
1	-x, -y, -z	11.67	-1.2	-3.1	-16.4	1.9	-16.5
1	-	6.71	-2.1	-1.2	-12.3	10.4	-5.6
2	x, y, z	13.95	-14.4	-3.5	-21.5	14.1	-24.9
1	-	11.91	-41.9	-6.0	-9.9	52.7	-12.7
1	-	5.06	-4.5	-0.4	-22	13.8	-13.4
1	-x, -y, -z	14.04	-1.8	-0.6	-6.2	0.6	-7.3
1	-	5.17	-5.1	-0.5	-20.5	14.6	-12.2
1	-	11.89	0.1	-0.2	-4.6	2.7	-2.0
1	-	11.85	-50	-7.1	-10.2	65.9	-11.3
1	-x, -y, -z	14.28	0.8	-0.5	-3	0.0	-2.2
1	-	12.45	0.4	-0.1	-2	0.2	-1.2
1	-x, -y, -z	10.85	1.1	-0.1	-1.4	0.0	-0.2
1	-x, -y, -z	10.93	1.3	-0.1	-1.6	0.0	-0.2
1	-x, -y, -z	15.32	1.5	-0.3	-2.1	0.0	-0.5
1	-x, -y, -z	13.66	-1.3	-0.2	-1.8	0.0	-3.1

Table S2: Interaction Energies (kJ/mol) calculated for **1-I_2** within a radius of 5.0 Å from each atom in **I_2**. R is the distance between molecular centroids (mean atomic position) in Å. Total energies, reported for the benchmarked HF/3-21G level, are the sum of the four energy components, scaled appropriately (k_ele=1.019, k_pol=0.651, k_disp=0.901, k_rep= 0.811).

N	Sym. Op.	R	E_ele	E_pol	E_dis	E_rep	E_tot
1	-	6.55	-3.5	-1.2	-12.6	10.4	-7.3
1	-	11.91	-41.9	-6.0	-9.9	52.7	-12.7
1	-	6.71	-2.1	-1.2	-12.3	10.4	-5.6
1	-	5.17	-5.1	-0.5	-20.5	14.6	-12.2
1	-	11.89	0.1	-0.2	-4.6	2.7	-2.0
1	-	5.06	-4.5	-0.4	-22.0	13.8	-13.4
1	-	11.85	-50.0	-7.1	-10.2	65.9	-11.3
1	-	12.45	0.4	-0.1	-2.0	0.2	-1.2
1	-x, -y, -z	4.61	-0.6	0.0	-9.2	5.6	-4.3
1	-x, -y, -z	4.66	-2.3	0.0	-10.1	6.1	-6.5

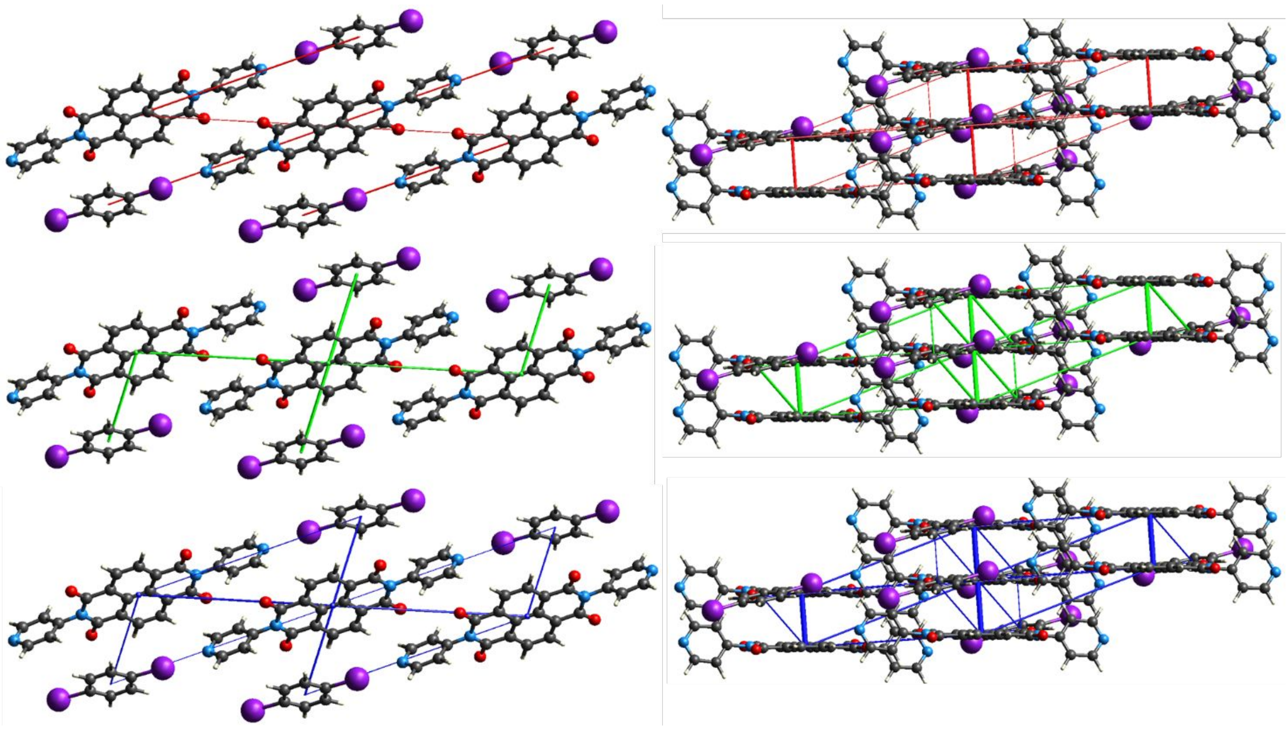

Figure S29: Energy decomposition of the main interactions for **1-DIB** (total=blue, dispersive=green, coulombic=red) in two different interactions. Lines thickness are proportional to the intensity of the interactions (threshold =-10 kJ/mol).

Table S3: Interaction Energies (kJ/mol) calculated for **1-DIB** within a radius of 5.0 Å from each atom in **1.** R is the distance between molecular centroids (mean atomic position) in Å. Total energies, reported for the benchmarked HF/3-21G level, are the sum of the four energy components, scaled appropriately (k_ele=1.019, k_pol=0.651, k_disp=0.901, k_rep= 0.811).

N	Sym. Op.	R	E_ele	E_pol	E_dis	E_rep	E_tot
2	-	5.48	-1.2	-2.8	-47.0	25.1	-25.1
2	x, y, z	5.38	-44.9	-10.3	-83.0	51.0	-85.9
2	x, y, z	11.57	-12.3	-3.5	-33.9	17.2	-31.5
2	x, y, z	13.73	0.4	-0.1	-1.7	0.0	-1.2
2	-	8.12	-9.6	-2.7	-15.1	11.3	-16.1
2	x, y, z	11.7	-4.4	-4.4	-21.6	5.0	-22.7
2	-	14.06	-23.0	-3.6	-9.8	29.1	-11.1
2	-	7.21	-2.1	-0.4	-7.4	1.6	-7.8
2	x, y, z	17.63	-4.3	-0.5	-3.5	0.4	-7.6
2	-	13.92	-0.7	-0.2	-3.1	0.5	-3.2

Table S4: Interaction Energies (kJ/mol) calculated for **1-DIB** within a radius of 5.0 Å from each atom in **DIB.** R is the distance between molecular centroids (mean atomic position) in Å. Total energies, reported for the benchmarked HF/3-21G level, are the sum of the four energy components, scaled appropriately (k_ele=1.019, k_pol=0.651, k_disp=0.901, k_rep= 0.811).

N	Sym. Op.	R	E_ele	E_pol	E_dis	E_rep	E_tot
2	-	14.06	-23.0	-3.6	-9.8	29.1	-11.1
2	-	8.12	-9.6	-2.7	-15.1	11.3	-16.1
2	-	5.48	-1.2	-2.8	-47.0	25.1	-25.1
2	-	7.21	-2.1	-0.4	-7.5	1.6	-7.8
2	x, y, z	5.38	-11.4	-1.3	-26.6	24.4	-16.6
2	-	13.92	-0.7	-0.2	-3.1	0.5	-3.2

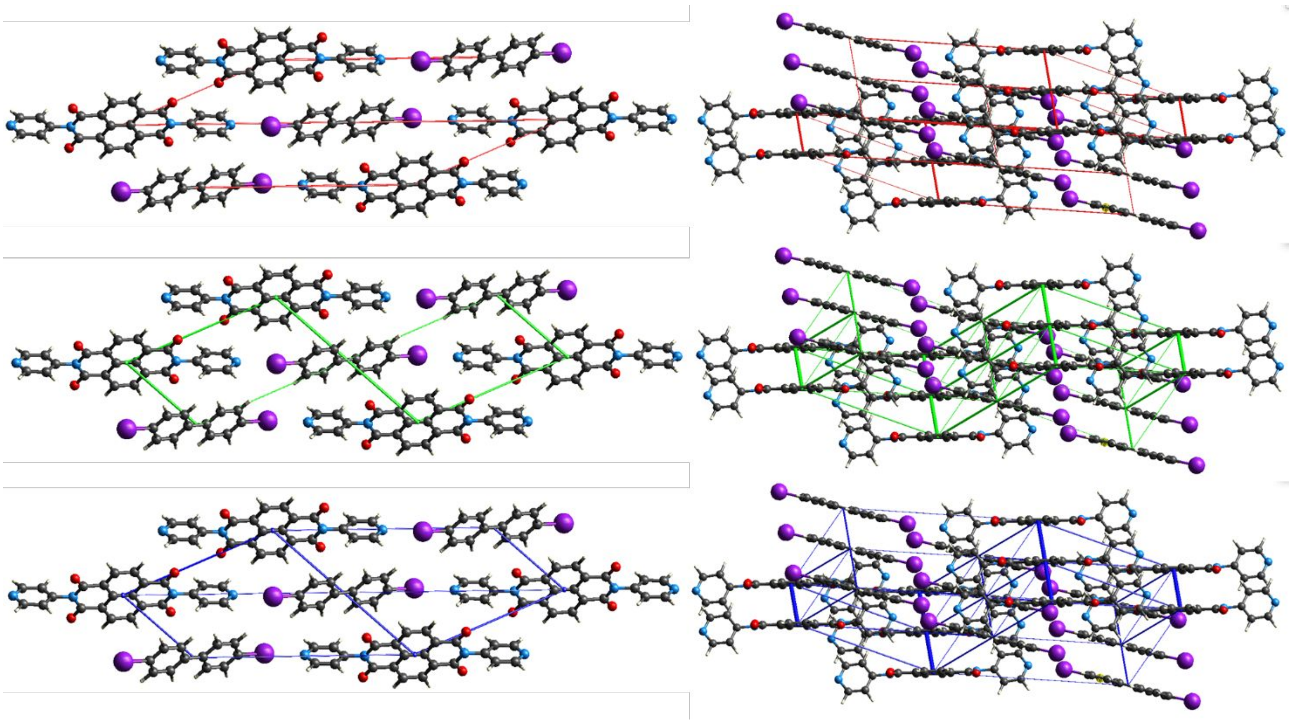

Figure S30: Energy decomposition of the main interactions for **1-DIBPH** (total=blue, dispersive=green, coulombic=red) in two different interactions. Lines thickness are proportional to the intensity of the interactions (threshold =-10 kJ/mol).

Table S5: Interaction Energies (kJ/mol) calculated for **1-DIBPH** within a radius of 5.0 Å from each atom in **1.** R is the distance between molecular centroids (mean atomic position) in Å. Total energies, reported for the benchmarked HF/3-21G level, are the sum of the four energy components, scaled appropriately (k_ele=1.019, k_pol=0.651, k_disp=0.901, k_rep= 0.811).

N	Sym. Op.	R	E_ele	E_pol	E_dis	E_rep	E_tot
2	x, y, z	5.33	-45.5	-10.7	-83.5	51.7	-86.7
2	x, y, z	11.77	-4.3	-4.7	-21.9	5.3	-22.9
2	-	7.5	-1.5	-0.9	-14.2	3.4	-12.2
2	-	6.87	-5.3	-4.0	-50.3	26.0	-32.2
2	x, y, z	11.77	-13.2	-3.6	-33.1	18.7	-30.5
2	x, y, z	13.97	0.4	-0.1	-1.6	0.0	-1.1
2	-	9.75	-9.1	-2.5	-14.6	10.2	-15.8
2	-	15.52	-0.3	-0.2	-3.4	0.8	-3.0
2	-	16.13	-21.8	-3.4	-9.7	27.5	-10.9

Table S6: Interaction Energies (kJ/mol) calculated for **1-DIBPH** within a radius of 5.0 Å from each atom in **DIBPH.** R is the distance between molecular centroids (mean atomic position) in Å. Total energies, reported for the benchmarked HF/3-21G level, are the sum of the four energy components, scaled appropriately (k_ele=1.019, k_pol=0.651, k_disp=0.901, k_rep= 0.811).

N	Sym. Op.	R	E_ele	E_pol	E_dis	E_rep	E_tot
2	-	6.87	-5.3	-4.0	-50.3	26.0	-32.2
2	-	16.13	-21.8	-3.4	-9.7	27.5	-10.9
2	-	15.52	-0.3	-0.2	-3.4	0.8	-3.0
2	x, y, z	11.77	-3.5	-0.5	-11.5	9.2	-6.8
2	x, y, z	11.77	-0.3	0.0	-1.0	0.0	-1.2
2	-	7.5	-1.5	-0.9	-14.2	3.4	-12.2
2	-	9.75	-9.1	-2.5	-14.6	10.2	-15.8
2	x, y, z	5.33	-14.3	-2.4	-45.5	27.7	-34.6
2	x, y, z	13.97	0.2	-0.1	-3.9	1.7	-2.0

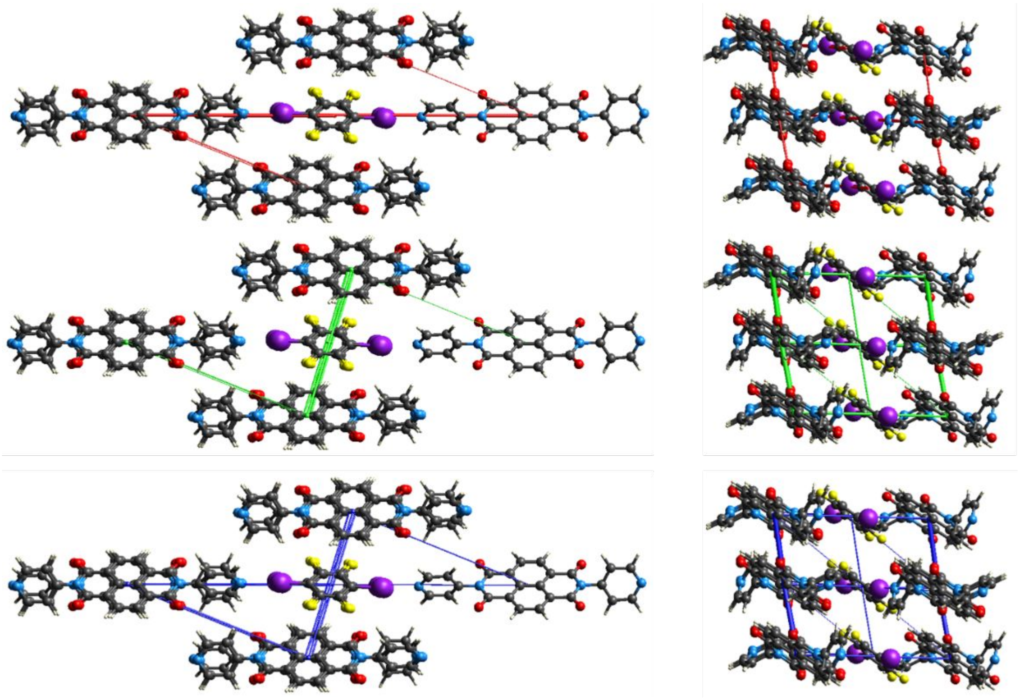

Figure S31: Energy decomposition of the main interactions for **1-DITFB** (total=blue, dispersive=green, coulombic=red) in two different interactions. Lines thickness are proportional to the intensity of the interactions (threshold =-10 kJ/mol).

Table S7: Interaction Energies (kJ/mol) calculated for **1-DITFB** within a radius of 5.0 Å from each atom in **1.** R is the distance between molecular centroids (mean atomic position) in Å. Total energies, reported for the benchmarked HF/3-21G level, are the sum of the four energy components, scaled appropriately (k_ele=1.019, k_pol=0.651, k_disp=0.901, k_rep= 0.811).

N	Sym. Op.	R	E_ele	E_pol	E_dis	E_rep	E_tot
1	-x, -y, -z	4.97	-31.7	-9.1	-91.3	46.0	-83.2
1	-	7.99	-4.5	-1.7	-13.1	6.8	-12.0
1	-x, -y, -z	16.49	-2.0	-0.4	-4.1	1.0	-5.2
1	-	6.52	0.8	-0.3	-6.5	0.1	-5.1
1	-	14.58	0.7	-0.1	-2.0	0.1	-1.0
1	-x, -y, -z	14.39	-0.6	-0.5	-4.9	0.4	-5.1
2	x, y, z	13.56	-14.6	-2.9	-14.0	8.0	-22.9
1	-x, -y, -z	4.90	-31.7	-9.6	-96.4	49.3	-85.5
1	-x, -y, -z	13.76	-0.4	-0.6	-5.8	0.4	-5.7
1	-	5.43	-8.7	-2.3	-47.7	24.4	-33.4
1	-x, -y, -z	17.12	-2.2	-0.3	-3.1	0.6	-4.8
2	x, y, z	16.39	-0.6	-0.5	-4.6	0.3	-4.9
1	-	6.62	0.9	-0.3	-6.9	0.3	-5.3
1	-	14.08	-47.1	-7.8	-10.1	50.6	-21.1
1	-	14.02	-53.4	-8.8	-10.5	61.2	-20.0
1	-	7.93	-6.0	-1.7	-14.1	6.7	-14.5
1	-	5.32	-8.1	-2.4	-48.5	25.8	-32.6
1	-	14.59	0.5	-0.1	-1.8	0.1	-1.2

Table S8: Interaction Energies (kJ/mol) calculated for **1-DITFB** within a radius of 5.0 Å from each atom in **DITFB.** R is the distance between molecular centroids (mean atomic position) in Å. Total energies, reported for the benchmarked HF/3-21G level, are the sum of the four energy components, scaled appropriately (k_ele=1.019, k_pol=0.651, k_disp=0.901, k_rep= 0.811).

N	Sym. Op.	R	E_ele	E_pol	E_dis	E_rep	E_tot
1	-	7.99	-4.5	-1.7	-13.1	6.8	-12.0
1	-	14.58	0.7	-0.1	-2.0	0.1	-1.0
1	-x, -y, -z	4.88	-2.6	-1.1	-34.8	12.1	-24.9
1	-	6.52	0.8	-0.3	-6.5	0.1	-5.1
1	-	5.32	-8.1	-2.4	-48.5	25.8	-32.6
1	-	14.08	-47.1	-7.8	-10.1	50.6	-21.1
1	-	6.62	0.9	-0.3	-6.9	0.3	-5.3
1	-	7.93	-6.0	-1.7	-14.1	6.7	-14.5
1	-	5.43	-8.7	-2.3	-47.7	24.4	-33.4
1	-	14.59	0.5	-0.1	-1.8	0.1	-1.2
1	-x, -y, -z	4.98	-2.8	-1.1	-32.1	9.5	-24.8
1	-	14.02	-53.4	-8.8	-10.5	61.2	-20.0

Full interaction map

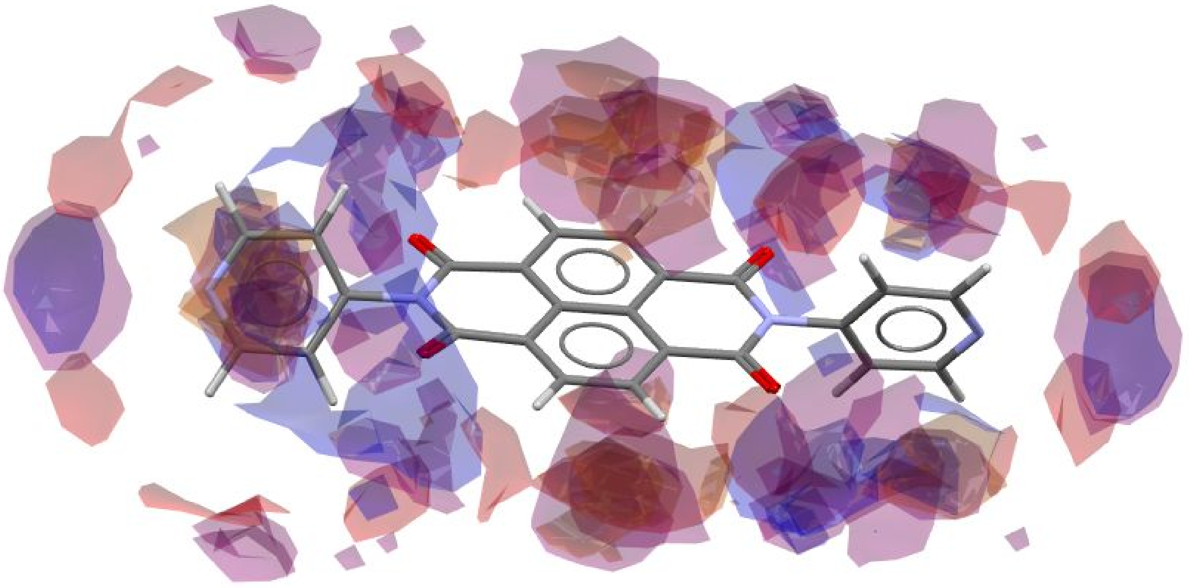

Figure S32: Full interaction map of the NDI molecule as observed in **1-I$_2$**

Geometrical description

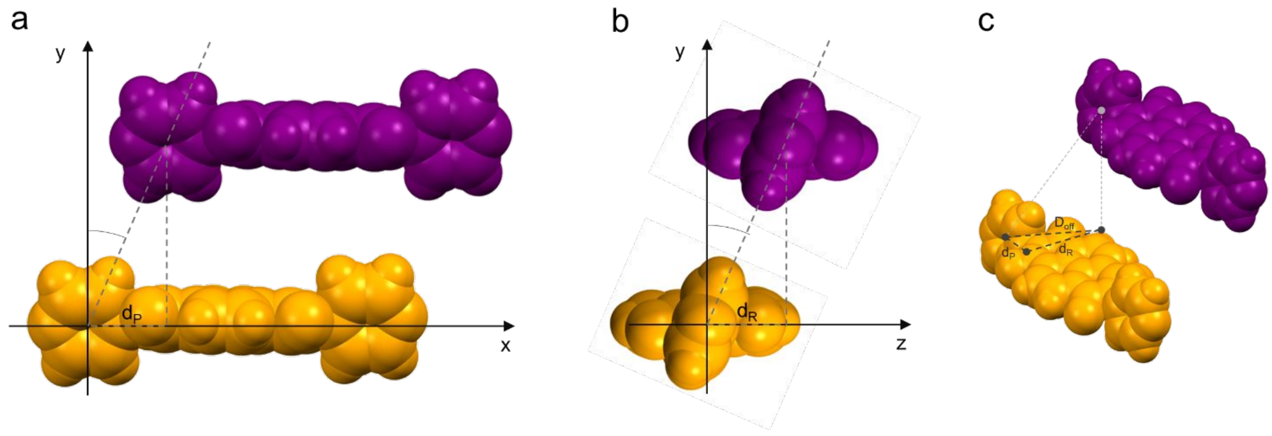

Figure S33: Geometrical schematization of pitch angle and pitch distance d_P (a), roll angle and roll distance d_R (b) and offset distance D_{off} (c) of a generic aromatic dimer interacting though π-π interaction.
